# The enrichment of Fanconi anemia/homologous recombination pathway aberrations in *ATM*/*ATR*-mutated NSCLC was accompanied by unique molecular features and poor prognosis

**DOI:** 10.1186/s12967-023-04634-1

**Published:** 2023-12-01

**Authors:** Wei Wei, Fangfang Shi, Yang Xu, Yang Jiao, Ying Zhang, Qiuxiang Ou, Xue Wu, Lingyi Yang, Jinhuo Lai

**Affiliations:** 1 Department of Oncology, The First Affiliated Hospital of Anhui Medical University, Hefei, 230022 Anhui China; 2 Department of Oncology, Zhongda Hospital Southeast University, Nanjing, 210009 Jiangsu China; 3 Geneseeq Research Institute, Nanjing Geneseeq Technology Inc., Nanjing, 210032 Jiangsu China; 4 Department of Pathology, Jinling Hospital, Nanjing University School of Medicine, Nanjing, 210002 Jiangsu China; 5 Department of Pulmonary and Critical Care Medicine, The First Affiliated Hospital of Soochow University, 188 Shizi Street, Suzhou, 215006 Jiangsu China; 6 Department of Medical Oncology, Fujian Medical University Union Hospital, 29 Xinquan Road, Fuzhou, 350025 Fujian China

**Keywords:** ATM, ATR, Fanconi anemia, Homologous recombination, Tumor mutational burden, Microsatellite instability, NSCLC

## Abstract

**Background:**

ATM and ATR are two critical factors to regulate DNA damage response (DDR), and their mutations were frequently observed in different types of cancer, including non-small cell lung cancer (NSCLC). Given that the majority of identified *ATM*/*ATR* mutations were variants of uncertain significance, the clinical/molecular features of pathogenic *ATM*/*ATR* aberrations have not been comprehensively investigated in NSCLC.

**Methods:**

Next-generation sequencing (NGS) analyses were conducted to investigate the molecular features in 191 NSCLC patients who harbored pathogenic/likely pathogenic *ATM*/*ATR* mutations and 308 NSCLC patients who did not have any types of *ATM*/*ATR* variants. The results were validated using an external cohort of 2727 NSCLC patients (including 48 with *ATM*/*ATR* pathogenic mutations).

**Results:**

Most pathogenic *ATM*/*ATR* genetic alterations were frameshift and nonsense mutations that disrupt critical domains of the two proteins. *ATM*/*ATR*-mutated patients had significantly higher tumor mutational burdens (TMB; *P* < 0.001) and microsatellite instabilities (MSI; *P* = 0.023), but not chromosomal instabilities, than those without any *ATM*/*ATR* variations. In particular, *KRAS* mutations were significantly enriched in *ATM*-mutated patients (*P* = 0.014), whereas *BRCA2* mutations (*P* = 0.014), *TP53* mutations (*P* = 0.014), and *ZNF703* amplification (*P* = 0.008) were enriched in *ATR*-mutated patients. Notably, patients with *ATM*/*ATR* pathogenic genetic alterations were likely to be accompanied by mutations in Fanconi anemia (FA) and homologous recombination (HR) pathways, which were confirmed using both the study (*P* < 0.001) and validation (*P* < 0.001) cohorts. Furthermore, the co-occurrence of FA/HR aberrations could contribute to increased TMB and MSI, and patients with both *ATM*/*ATR* and FA/HR mutations tended to have worse overall survival.

**Conclusions:**

Our results demonstrated the unique clinical and molecular features of pathogenic *ATM*/*ATR* mutations in NSCLC, which helps better understand the cancerous involvement of these DDR regulators, as well as directing targeted therapies and/or immunotherapies to treat *ATM*/*ATR*-mutated NSCLC, especially those with co-existing FA/HR aberrations.

**Supplementary Information:**

The online version contains supplementary material available at 10.1186/s12967-023-04634-1.

## Background

Normal cells are persistently exposed to exogenous and endogenous challenges that induce DNA damage, and DNA damage response (DDR) pathways help maintain the integrity of the genome and prevent neoplasia, as DDR is closely associated with cell cycle arrest, DNA repair, and apoptotic machinery [1]. As a result, genomic instability caused by DDR deficits is one of the key hallmarks of cancer, given that DDR is critical for both tumorigenesis and cancer treatment [2]. Ataxia telangiectasia mutated protein (ATM) and Ataxia telangiectasia and Rad3 related protein (ATR), which belong to the phosphatidylinositol 3-kinase-related kinase (PIKK) family, are two of the major regulators of DDR [3]. Indeed, more than 4000 cancer-associated *ATM*/*ATR* mutations were identified in various types of cancer, including lung cancer, breast cancer, colorectal cancer, pancreatic cancer, prostate cancer, and endometrial cancer, although the function and cancer implications of most of these *ATM*/*ATR* mutations were largely unknown [4]. Therefore, it is clinically important to characterize pathogenic *ATM*/*ATR* mutations, which would in turn help infer the involvement of other variants of uncertain significance of these two PIKK genes in cancer.

Previous studies have found the close interplay among different DDR pathways, and the deficiency in one DDR factor leads to heavy dependence on other DDR components in order to maintain viability during DNA damage [5]. This provides a promising strategy to target cancer. Besides the well-known poly(ADP-ribose) polymerase (PARP) inhibitors to target *BRCA1/2*-deficient cancers [6], preclinical studies have shown that defects in *ATM*, *BRCA1/2*, and *TP53* confer sensitivity to ATR inhibition in tumor cells [7–10]. Thereby, multiple different ATM and ATR inhibitors have been developed, many of which are currently under clinical investigation [11, 12]. For example, Berzosertib is a highly potent and first-in-class ATR inhibitor that has demonstrated promising results during phases I and/or II clinical trials against non-small cell lung cancer (NSCLC) [13], small cell lung cancer [14], triple-negative breast cancer [15], high-grade serous ovarian cancer [16], and advanced solid tumors [17, 18]. Several other ATR inhibitors (e.g., Ceralasertib, M4344, and Elimusertib) and ATM inhibitors (e.g., AZD0156, AZD1390, KU60019, M3541, and M4076) are also being actively studied [19–25]. As a result, it is imperative to identify a subset of patients who may benefit from these newly developed ATM/ATR inhibitor drugs.

In the current study, we retrospectively investigated 191 NSCLC patients with pathogenic or likely pathogenic *ATM*/*ATR* mutations using large-panel next-generation sequencing (NGS), and the result was then compared with that of 308 NSCLC patients without any types of *ATM*/*ATR* variants to elucidate the PIKK aberration-associated clinical and genomic features. Our conclusions were then further validated using an external cohort of 2727 NSCLC patients (48 with pathogenic/likely pathogenic *ATM*/*ATR* mutations and 2679 without any *ATM*/*ATR* variations) from publicly available NGS databases. We aimed to demonstrate the unique molecular patterns associated with *ATM*/*ATR* alterations in NSCLC and stratify patients for targeted therapy.

## Methods

### Patients and sample collection

A total of 191 NSCLC patients who harbored pathogenic or likely pathogenic *ATM*/*ATR* mutations, which were identified using ClinVar (https://www.ncbi.nlm.nih.gov/clinvar/), as well as NSCLC patients with frameshift/nonsense mutations that disrupt critical domain(s) of ATM/ATR, were recruited from the First Affiliated Hospital of Anhui Medical University, Zhongda Hospital Southeast University, The First Affiliated Hospital of Soochow University, and Fujian Medical University Union Hospital. A randomly selected cohort of 308 NSCLC patients without any types of *ATM*/*ATR* variants was recruited from the same hospitals (Additional file 1: Fig S1). Baseline tumor samples were collected from each patient and underwent broad-panel NGS of 425 cancer-relevant genes (GENESEEQPRIME^™^, Geneseeq Technology Inc.) [26]. This study was approved by Medical Ethics Committee of Nanjing Geneseeq Medical Laboratory, and all patients provided written informed consent to participate and publication. Qualified NGS data of 2727 NSCLC patients, including 48 patients with pathogenic *ATM*/*ATR* mutations and 2679 without any types of *ATM*/*ATR* variants, were obtained from cBioPortal (http://www.cbioportal.org) and used as an external cohort to validate our results.

### DNA extraction and library preparation

DNA extraction, quantification, and library preparation were performed as previously described [27]. Briefly, formalin-fixed paraffin-embedded (FFPE) samples were de-paraffinized with xylene and DNA was extracted using the QIAamp DNA FFPE Tissue Kit (Qiagen) according to the manufacturer’s instructions. Genomic DNA from fresh tumor tissue was extracted using the DNeasy Blood & Tissue Kit (Qiagen) according to the manufacturer’s protocols. Peripheral blood samples were centrifuged at 1800 g for 10 min, and the genomic DNA from white blood cells was extracted using DNeasy Blood and Tissue Kit (Qiagen). Genomic DNA was qualified using a Nanodrop2000 (Thermo Fisher Scientific, Waltham, MA). All DNA was quantified using the dsDNA HS assay kit on a Qubit 3.0 fluorometer (Life Technology, US) according to the manufacturer’s recommendations. Sequencing libraries were prepared using the KAPA Hyper Prep kit (KAPA Biosystems) with an optimized manufacturer’s protocol and sequenced as previously described [27].

### NGS data processing

Sequencing data were processed as previously described [27]. In brief, the data was first demultiplexed and subjected to FASTQ file quality control to remove low-quality data or N bases. Qualified reads were mapped to the reference human genome hg19 using Burrows-Wheller Aligner and Genome Analysis Toolkit (GATK 3.4.0) was employed to apply the local realignment around indels and base quality score recalibration. Picard was used to remove PCR duplicates. VarScan2 was employed for the detection of single-nucleotide variations (SNVs) and insertion/deletion mutations. SNVs were filtered out if the mutant allele frequency (MAF) was less than 1%. Common SNVs were excluded if they were present in > 1% population in the 1000 Genomes Project or the Exome Aggregation Consortium (ExAC) 65,000 exomes database. The resulting mutation list was further filtered by an in-house list of recurrent artifacts based on a normal pool of whole blood samples. Parallel sequencing of matched white blood cells from each patient was performed to further remove sequencing artifacts, germline variants, and clonal hematopoiesis. The copy number alterations were analyzed as previously described [28, 29]. The tumor purities were first estimated using ABSOLUTE [30]. Somatic copy number alteration events were assigned based on sample-ploidy values calculated in the FACETS algorithm [31]. Loss-of-heterozygosity (LOH) was also calculated using FACETS and determined using the minor copy number estimates of each segment for genes in the targeted panel. The minor copy number is by definition 0 in a LOH event [32, 33]. Structural variants were detected using FACTERA with default parameters [34]. The fusion reads were further manually reviewed and confirmed on Integrative Genomics Viewer.

Tumor mutational burden (TMB, mutation per Megabase) was determined based on the number of somatic base substitutions and indels in the targeted regions of the gene panel covering 0.85 Mb of coding genome, excluding known driver mutations as they are over-represented in the panel. Chromosome instability score (CIS) was defined as the proportion of the genome with aberrant (purity-adjusted segment-level copy number ≥ 3 or ≤ 1) segmented copy number [35].

### Immune infiltration analysis

The association among *ATM*/*ATR* mutations, histological subtypes, and tumor immune infiltration was analyzed using Timer 2.0 (http://timer.cistrome.org). The results were based on the gene mutation and expression data of 515 lung adenocarcinoma (ADC) and 485 lung squamous cell carcinoma (SCC) patients from The Cancer Genome Atlas (TCGA) database (https://www.cancer.gov/about-nci/organization/ccg/research/structural-genomics/tcga).

### Statistical analysis

The comparison of the proportion of categorical variables was done using Fisher’s exact test, and the comparison between groups of quantitative variables was performed by Welch’s t-test. All the multiple comparisons were corrected by the Benjamini & Hochberg approach. Kyoto encyclopedia of genes and genomes (KEGG) pathway over-representation analysis (ORA) and gene ontology (GO) enrichment analysis were conducted using the clusterProfiler (v4.4.4) R package [36]. The co-occurring and mutually exclusive analysis was performed using the maftools R package. Kaplan–Meier survival curve was used to analyze the overall survival (OS) of various patient groups, and the statistical difference was analyzed using the log‐rank test (analyzed using the survival R package). Two-sided *P* values of less than 0.05 were considered statistically significant. Statistical analyses were performed using the R (v4.2.1).

## Results

### Patient characteristics of the study and external patient cohorts

A total of 191 NSCLC patients with pathogenic or likely pathogenic *ATM*/*ATR* mutations (denoted as “PIKK-mutated”) were included in the study cohort, and only one patient (P145) harbored both *ATM* and *ATR* pathogenic mutations (Additional file 1: Table S1). We also included 308 NSCLC patients without any types of *ATM*/*ATR* variants (denoted as “PIKK-wild type/WT”), and all 499 patients were grouped as a study cohort and their baseline tumor samples underwent broad-panel NGS of 425 cancer-relevant genes to obtain their mutational profile, tumor mutational burden (TMB), copy-number variation (CNV), CIS, and microsatellite instability (MSI) status (Additional file 1: Fig S1). For the 191 PIKK-mutated patients, the median age was 64 years (ranging from 27 to 89) and more patients were males (72.8%); for the 308 PIKK-WT patients, the median age was 60 years (ranging from 28 to 89) and less proportion of patients were males when compared with those with positive PIKK mutations (*P* < 0.001; Additional file 1: Table S2). The majority of the histological subtype was ADC, which is comparable between patients with and without PIKK mutations (*P* = 0.773; Additional file 1: Table S2).

To validate the results obtained from the study cohort, we obtained NGS data of 2727 NSCLC patients from cBioPortal database and grouped them as an external cohort, which comprised 48 patients with pathogenic or likely pathogenic *ATM*/*ATR* mutations (Additional file 1: Table S3) and 2679 patients without any *ATM*/*ATR* variations (Additional file 1: Fig S1). The median age for PIKK-mutated and PIKK-WT patients was 65 and 67 years, respectively, and both subgroups had a higher proportion of female patients; the histological subtypes between the two subgroups were also consistent (*P* = 0.543; Additional file 1: Table S4).

### PIKK pathogenic mutations were accompanied by high mutational loads and microsatellite instabilities

**Fig. 1:**
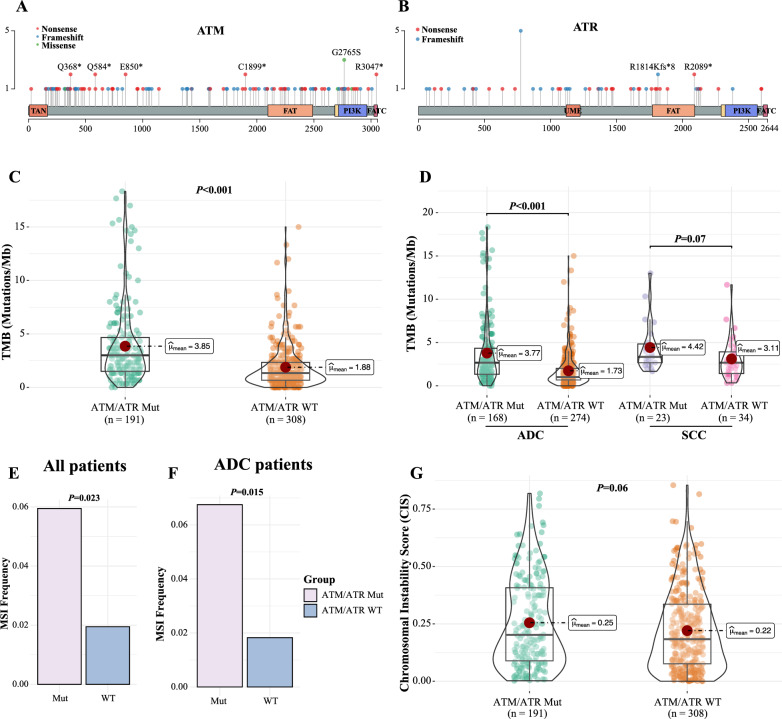
PIKK pathogenic mutations were accompanied by high mutational loads and microsatellite instabilities. **A**, **B** The lollipop plots of the pathogenic mutations of *ATM* (**A**) and *ATR* (**B**) detected in the study cohort. (**C**) The tumor mutational burden (TMB) in PIKK-mutated (Mut) and PIKK-wild type (WT) NSCLC patients from the study cohort. **D** The comparison of TMB between different PIKK mutational statuses and histological subtypes in the study cohort. **E**, **F** The comparison of microsatellite instability (MSI) between PIKK-mutated and PIKK-WT NSCLC patients for all patients (**E**) or ADC patients (**F**) from the study cohort. **G** The chromosomal instability score (CIS) between PIKK-mutated and PIKK-WT NSCLC patients from the study cohort

As shown in Fig. 1A, B, the majority of the pathogenic or likely pathogenic *ATM*/*ATR* genetic alterations were frameshift and nonsense mutations, most of which were located before critical domains of these two PIKK proteins. We, thereby, compared the molecular features between patients with mutated (Mut) or wild-type (WT) PIKK. Strikingly, PIKK aberrations were significantly associated with higher TMB in the 499 NSCLC patients from the study cohort (*P* < 0.001, Fig. 1C), and increased mutational loads were observed for both ADC and SCC histological subtypes (Fig. 1D). These results were then confirmed using the external NSCLC cohort (Additional file 1: Fig S2A, B). Consistent with the elevated mutational burdens, PIKK-positive patients also had higher microsatellite instabilities than PIKK-negative patients (*P* = 0.023, Fig. 1E), and this observation was more significant in ADC subtypes (*P* = 0.015, Fig. 1F) but not SCC subtypes due to limited cohort size (Additional file 1: Fig S2C). In contrast, PIKK mutations seemed not to have profound impacts on chromosomal instability in these lung cancer patients (Fig. 1G and Additional file 1: Fig S2D). Therefore, PIKK pathogenic alterations were likely to associate with nucleotide level rather than chromosomal level changes in NSCLC.

### Unique genetic profiles in PIKK-mutated patients

**Fig. 2:**
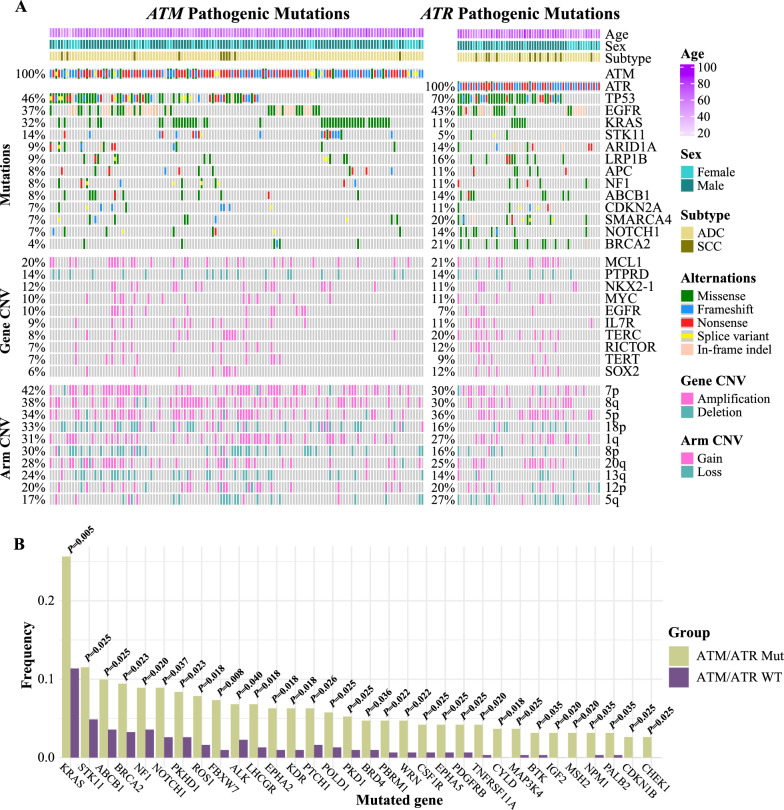
The distinct genetic profile in PIKK-mutated patients. **A** The oncoprint plot of the genetic profile for NSCLC patients with either *ATM* or *ATR* pathogenic mutations from the study cohort. The patient (P145) who harbored both *ATM* and *ATR* pathogenic mutations was not included. **B** The mutated genes that were significantly enriched in PIKK-mutated patients from the study cohort. The analyses were done using Fisher’s exact test, and all multiple comparisons were corrected by the Benjamini & Hochberg approach

Given that PIKK pathogenic alterations might mainly induce mutation-level changes, we investigated the genetic profiles in patients with different PIKK mutational statuses. Intriguingly, *TP53*, *EGFR*, and *KRAS* were the most frequently mutated genes in both PIKK-mutated and PIKK-WT patients (Fig. 2A and Additional file 1: Fig S2E). Nevertheless, the PIKK-mutated group had a significantly higher frequency of *KRAS* mutations than the PIKK-WT group (*P* = 0.005, Fig. 2B). Indeed, almost all genes with significantly higher mutational frequency came from the PIKK-mutated group, such as *STK11* (11.5% vs 4.9%, *P* = 0.025), *BRCA2* (9.4% vs 3.2%, *P* = 0.023), *NF1* (8.9% vs 3.6%, *P* = 0.020), *ROS1* (7.9% vs 1.6%, *P* = 0.018), and *FBXW7* (7.3% vs 0.9%, *P* = 0.008) (Fig. 2B). On the other hand, although a large number of gene-level and arm-level CNVs were identified, including the amplification of *MCL1*, *NKX2-1*, *MYC*, and *EGFR*, the gain of arms 7p/8q/5p, and the deletion of *PTPRD* and arm 18p (Fig. 2A), only arms 8q and 15q CNVs were significantly enriched in PIKK-mutated patients (Additional file 1: Fig S3A) while no gene-level CNV was significantly differentially altered after correcting for multiple comparisons (Additional file 1: Fig S3B).

### Fanconi anemia and homologous recombination pathway aberrations were enriched in PIKK-mutated patients

**Fig. 3:**
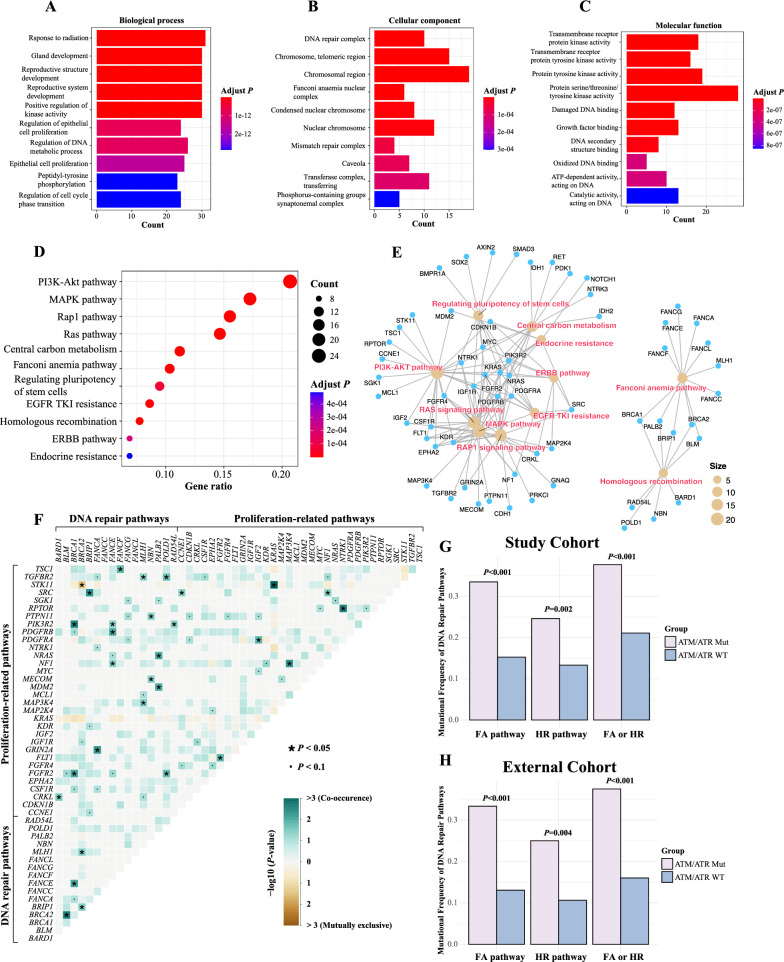
Fanconi anemia (FA) and homologous recombination (HR) pathway aberrations were enriched in PIKK-mutated NSCLC patients. **A**–**C** The gene ontology analysis for mutations enriched in PIKK-mutated patients than PIKK-WT patients from the study cohort. **D**, **E** The KEGG pathway analysis for mutations enriched in PIKK-mutated patients than PIKK-WT patients from the study cohort. **F** The analysis of co-occurring and mutually exclusive mutated genes in PIKK-mutated NSCLC patients in the study cohort. **G**, **H**) The enrichment of FA/HR pathway-related mutations in NSCLC patients with different PIKK mutational statuses for the study cohort (**G**) and the external cohort (**H**)

Next, we want to investigate the GO and pathway features in PIKK-mutated NSCLC. We included PIKK-enriched genetic changes with both the odds ratio and the number of mutated genes being at least 2 (Additional file 1: Table S5) to perform GO and pathway enrichment analyses. In particular, the GO enrichment results revealed that the cell proliferation and cell cycle regulation processes (Fig. 3A), the DNA repair-associated cellular components (Fig. 3B), and kinase activity-related molecular functions (Fig. 3C) were specifically enriched in PIKK-mutated patients. Based on KEGG pathway analyses, we discovered that multiple tumorigenesis, tumor progression, and cancer maintenance-associated signaling pathways were at the top of the list (Fig. 3D). Notably, those PIKK pathogenic mutation-enriched pathways could be clustered into 2 categories, one was proliferation-related pathways (e.g., RAS pathway, MAPK pathway, ERBB pathway, and PI3K-AKT pathway) and the other was DNA repair-related pathways, that is, Fanconi anemia (FA) and homologous recombination (HR) pathways (Fig. 3E). Moreover, we performed co-occurring and mutually exclusive analyses between the two categories of pathways that were detected in PIKK-mutated NSCLC patients (Fig. 3E). Only one pair of genes, *STK11* and *BRCA2*, were found to be mutually exclusive; in contrast, a large number of proliferation-promoting mutations co-occurred with DNA repair gene mutations, for example, *PDGFRB* and *FANCE* mutations, *BRIP1* and *SRC* mutations, *PIK3R2* and *BRCA1* mutations, *TGFBR2* and *POLD1* mutations, and *CRKL* and *BARD1* mutations (Fig. 3E).

### FA/HR aberrations were associated with higher nucleotide-level changes and worse prognosis in PIKK-mutated patients

**Fig. 4:**
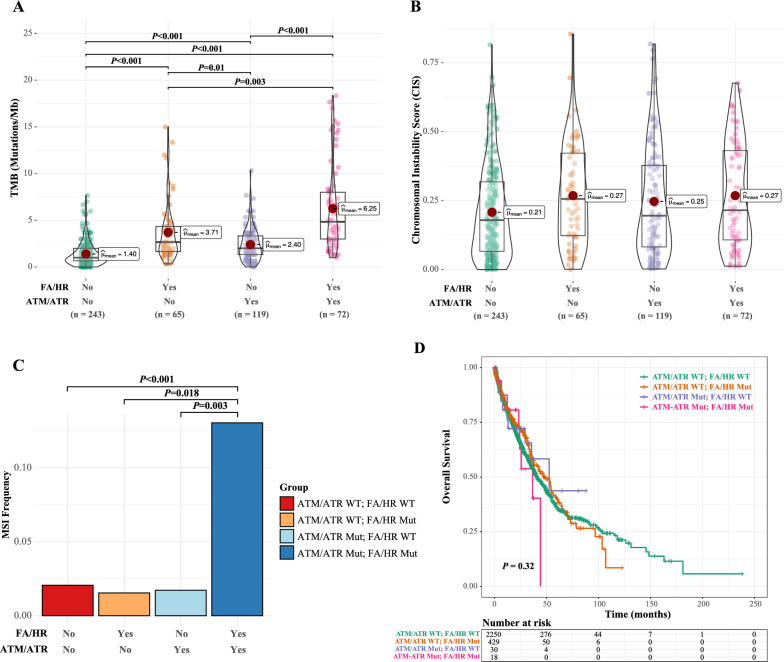
FA/HR aberrations were associated with higher nucleotide-level changes and worse prognosis in PIKK-mutated patients. **A** The comparison of TMB in NSCLC patients with different PIKK and FA/HR mutational statuses in the study cohort. **B** The comparison of CIS in NSCLC patients with different PIKK and FA/HR mutational statuses in the study cohort. **C** The comparison of MSI frequency in NSCLC patients with different PIKK and FA/HR mutational statuses in the study cohort. **D** Kaplan–Meier curve of overall survival in NSCLC patients from the external cohort in strata of different PIKK and FA/HR mutational statuses

Given that PIKK is closely related to DNA repair pathways, we then further investigated the molecular and functional involvement of FA and HR mutations in PIKK-mutated patients. Consistent with the above analysis, both FA and HR pathway aberrations were significantly enriched in PIKK-mutated patients in the study cohort (Fig. 3G), which was further validated using the external cohort (Fig. 3H). Additionally, considering the demographic differences in our patient cohorts (Additional file 1: Tables S2, S4), we found that age, sex, and histological subtypes did not have significant impacts on FA/HR enrichment in PIKK-mutated patients (Additional file 1: Fig S4). Intriguingly, as PIKK mutations were associated with increased TMB than PIKK-WT samples, the concurrent FA/HR mutations further elevated the mutational loads (Fig. 4A and Additional file 1: Fig S5A). Similarly, FA/HR aberrations, together with PIKK pathogenic mutations, were associated with the highest microsatellite instability but not chromosomal instability (Fig. 4B, C, Additional file 1: Fig S5B, C, S6A–F). The TMB results were further confirmed using the external cohort (Additional file 1: Fig S6G–I). Notably, there was a trend that patients with both PIKK and FA/HR mutations were correlated with poor overall survival for NSCLC patients (Fig. 4D and Additional file 1: Fig S6J, K). Therefore, the two mutated PIKK genes specifically co-occurred with FA/HR aberrations, which is likely to result in higher levels of nucleotide-level changes and poor prognosis in NSCLC.

### ATR mutations tended to associate with higher TMB, more MSI, and poorer prognosis than ATM mutations

**Fig. 5:**
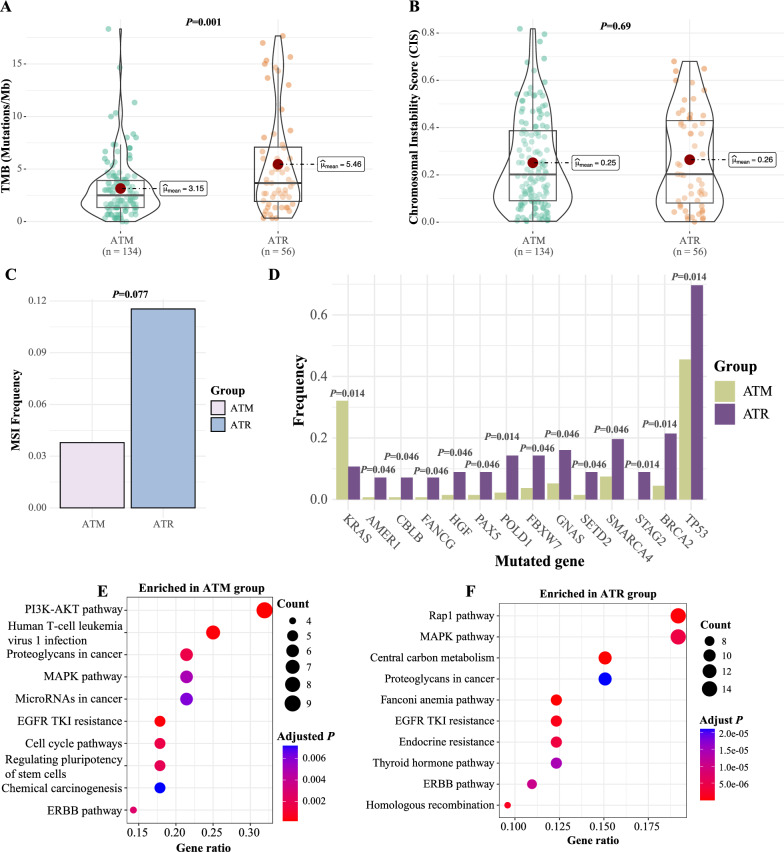
The unique genetic profile in *ATM*-mutated versus *ATR*-mutated patients. **A**–**C** The comparison of TMB (**A**), CIS (**B**), and MSI frequency (**C**) between *ATM*- and *ATR*-mutated patients from the study cohort. (**D**) The differentially mutated genes in *ATM*- and *ATR*-mutated patients from the study cohort. The analyses were done using Fisher’s exact test, and all multiple comparisons were corrected by the Benjamini & Hochberg approach. **E**, **F** The KEGG pathway analysis for mutated genes that were enriched in *ATM*-mutated patients (**E**) or *ATR*-mutated patients (**F**)

Lastly, we investigated *ATM* and *ATR* pathogenic mutations separately. Notably, although both *ATM*- and *ATR*-mutated patients had higher TMB than PIKK-WT patients (Fig. 1C), *ATR* was associated with significantly higher mutational burdens than *ATM* (Fig. 5A). Consistently, *ATR*-mutated patients tended to have higher MSI than *ATM*-mutated patients, while the two groups of patients had comparable chromosomal instability (Fig. 5B, C). By comparing the genetic profile between *ATM* and *ATR* mutational groups (Fig. 2A), *KRAS* mutations were significantly enriched in *ATM*-mutated patients (*P* = 0.014); in contrast, more genetic alterations, including *BRCA2* mutations (*P* = 0.014), *TP53* mutations (*P* = 0.014), and *ZNF703* amplification (*P* = 0.008), were enriched in *ATR*-mutated patients (Fig. 5D and Additional file 1: Fig S7A, B). Based on pathway analyses, many pathway aberrations were identified in both ATM and ATR groups, although the PI3K-AKT pathway was more enriched in *ATM*-mutated patients while DNA repair-related pathways were more enriched in *ATR*-mutated patients (Fig. 5E, F, Additional file 1: Fig S7C, D, Table S6).

**Fig. 6:**
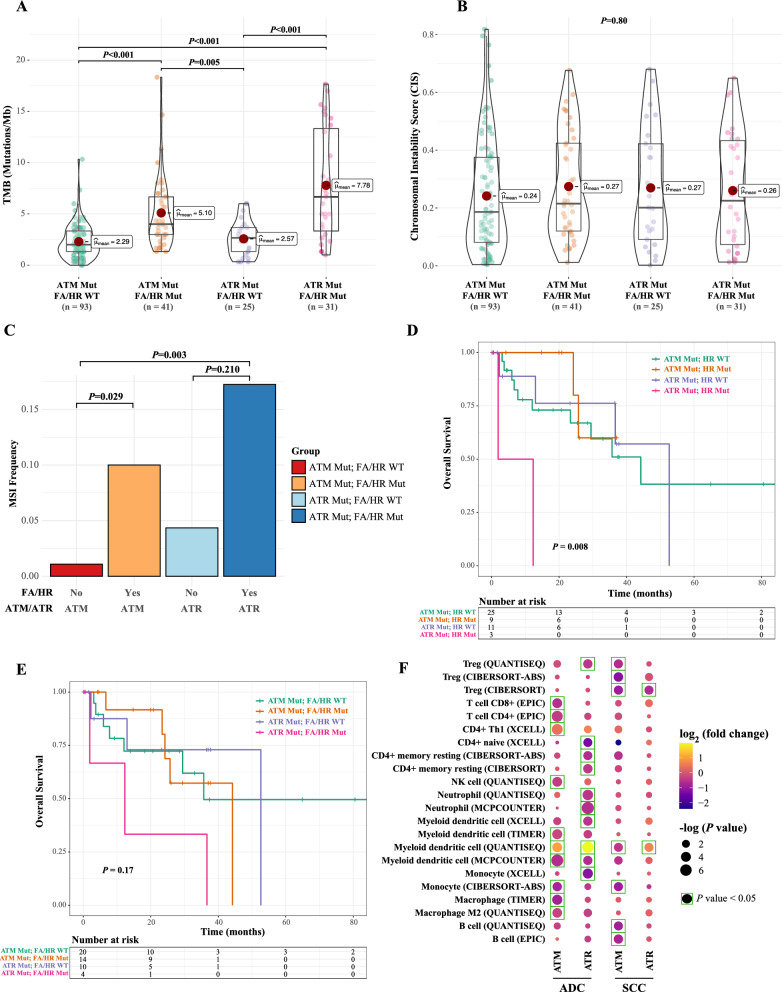
*ATR* and FA/HR co-mutations were specifically associated with more nucleotide-level changes and poor prognosis. **A**–**C** The comparison of TMB (**A**), CIS (**B**), and MSI frequency (**C**) in patients from the study cohort who were stratified by different *ATM*/*ATR* and FA/HR mutational statuses. **D** Kaplan–Meier curve of overall survival in NSCLC patients from the external cohort in strata of different *ATM*/*ATR* and HR mutational statuses. **E** Kaplan–Meier curve of overall survival in NSCLC patients from the external cohort in strata of different *ATM*/*ATR* and FA/HR mutational statuses. **F** The immune infiltration level stratified by different ATM/ATR mutational statuses and histological subtypes. The results were analyzed using Timer 2.0, which is based on the gene mutation and expression data from the TCGA database

We then tested the effects of concurrent FA/HR aberration on the *ATM*- or *ATR*-mutated group. Similar to the results of the PIKK group, both *ATM* and *ATR* mutated patients harbored more TMB and MSI, but not chromosomal instability, when they also carried FA/HR mutations (Fig. 6A–C and Additional file 1: Figure S8A–I). Interestingly, only *ATR* plus FA/HR mutations were associated with worse overall survival in NSCLC patients (Fig. 6D, E and Additional file 1: Fig S8J). To better understand the differential prognosis between *ATM*- or *ATR*-mutated patients, we investigated the immune cell infiltration stratified by PIKK mutational status and histological subtypes. As different algorithms were used to predict immune infiltration, we searched for those with consistent predictions by at least two algorithms. Specifically, *ATM*-mutated ADC patients had lower macrophage (especially the M2 macrophage) infiltration while *ATM*-mutated SCC patients had less B cell and regulatory T cell (Treg) infiltrations; additionally, *ATR*-mutated ADC patients were associated with less CD4 + T cell and neutrophil infiltrations (Fig. 6F). Therefore, the differential immune infiltration associated with different PIKK mutational statuses might partially explain the poor clinical outcomes associated with *ATR*-mutated NSCLC patients.

## Discussion

The DDR pathways are promising targets for anti-cancer therapy, and various DDR-related genes are currently under clinical development, such as *PARP*, *ATM*, *ATR*, *DNA-PK*, *CHK1*, *CHK2*, and *WEE1* [37]. Our study focused on the pathogenic mutations of *ATM* and *ATR*, as both genes are from the PIKK family that is critically involved in DNA damage repair in normal cells as well as tumor cells. Intriguingly, we found that NSCLC patients with either of these two PIKK gene alterations were significantly associated with higher TMB and MSI, but not chromosomal instabilities. This result is consistent with the cellular function of ATM/ATR, given that ATM primarily regulates the DNA double-strand breaks response while ATR is mainly activated by extensive single-strand DNA structures that result from stalled DNA replication forks [38]. Two major categories of signaling pathways were enriched in patients with *ATM*/*ATR* mutations, including proliferation-related and DNA repair-related pathways. In particular, *ATM*/*ATR*-mutated patients have a profound enrichment of FA and HR pathway aberrations, and these NSCLC patients had more mutational burdens, higher MSI, and worse prognosis. To the best of our knowledge, this is the largest study that analyzes the molecular and clinical implications of pathogenic *ATM*/*ATR* mutations in NSCLC.

DDR-related PIKK genes are commonly mutated in cancer [39]. Waskiewicz et al. reported 2551 *ATM* mutations and 1,394 *ATR* mutations from 46,588 tumor samples [4]. Notably, most of these mutations were variants of uncertain significance (VUS), and only a few of them were located at the kinase active site. As a result, it would be difficult to infer the clinical characteristics and implications of loss-of-function mutations of *ATM*/*ATR* in cancer patients. We, thereby, focused on the pathogenic and likely pathogenic *ATM*/*ATR* mutations, most of which were frameshift and nonsense changes that disrupted critical domains of these proteins. The unique genetic profiles and features that were associated with known pathogenic *ATM*/*ATR* mutations could then be used to predict the function of *ATM*/*ATR* VUS in future studies. Additionally, we found that *ATM*/*ATR* pathogenic mutations were frequently accompanied by FA/HR mutations in NSCLC patients, which is not observed in the non-*ATM*/*ATR* counterparts. This might imply a potential tumorigenesis process driven by detrimental DDR alterations in NSCLC. That is, the acquisition of one copy of *ATM*/*ATR* pathogenic mutation in pre-cancerous cells might not be sufficient to drive NSCLC tumor formation, and it also requires aberrations in FA/HR to disrupt the DDR pathways, thus further elevating the mutational rate and increasing the chance to introduce other crucial oncogenic mutations (Additional file 1: Fig S9). Consistent with this speculation, we observed that patients with both *ATM*/*ATR* and FA/HR mutations had the highest level of TMB and MSI, and they were also likely to harbor mutations in various proliferation-related pathways. Therefore, it is possible that the concurrent FA/HR aberrations and *ATM*/*ATR* mutations help fine-tune the DDR machinery to introduce enough mutations to drive tumorigenesis/tumor progression while keeping certain levels of genomic integrity to maintain tumor cell survival.

It has been shown that ATM and ATR regulate partially overlapped but nonredundant downstream pathways during DNA repair [40], and defects in one PIKK protein might be compensated by the other to maintain the survival of cancer cells [12]. For example, loss of ATM in tumors makes tumors more rely on ATR-mediated intra-S and G2/M checkpoints [41], and inhibition of ATR could selectively kill cancer cells with *ATM* deficiency [7]. Although most of the NSCLC patients in our cohort harbored only one pathogenic *ATM*/*ATR* mutation, the additional FA/HR mutations could further weaken the DDR pathways, making them vulnerable to further DDR inhibition. Multiple previous pre-clinical and clinical studies have investigated the use of specific DDR inhibitors to target tumors with some DDR mutations/deficiencies. In a phase 1b clinical trial, Plummer et al. explored the effects of berzosertib in advanced NSCLC with different DDR mutational statuses [13]. Intriguingly, they found that TMB levels, rather than the mutation of a single DDR gene, were better associated with the response to berzosertib, and patients with high TMB tended to have an improved objective response rate. Furthermore, Schram et al. conducted a phase 2b trial to treat patients with *BRCA1*/2- or *ATM*-altered advanced solid tumors using avelumab (an anti-PD-L1 monoclonal antibody) plus talazoparib (a PARP inhibitor) [42]. Although the trial did not meet the prespecified objective response rate, they found that the drug response rate in TMB-high patients was higher than in TMB-low patients. We discovered that *ATM*/*ATR*-mutated, FA/HR-altered patients had significantly higher TMB/MSI and poor prognosis. The FA pathway could repair DNA interstrand crosslinks [43], whereas the HR pathway is well known to regulate DNA double-strand break repair [44]. Cai’s group found that knockout of both *ATM* and genes in the FA pathway inhibited end resection and induced toxic levels of non-homologous DNA end joining, and FA-deficient tumors were sensitive to ATM inhibitors [45]. The dual DDR mutations in *ATM*/*ATR*-mutated, FA/HR-altered tumors were likely to make them susceptible to further DDR inhibition while high TMB/MSI are predictive markers for better response to immune checkpoint inhibitors (ICIs) [46]. Therefore, this sub-cohort of NSCLC might benefit from DDR inhibitor and/or immunotherapy treatments.

There were several limitations of our study. Firstly, as the clinical outcome of most of the patients were not available, we cannot perform further prognostic analyses. Secondly, although DNA-PK is another major DDR-related PIKK, we were not able to recruit enough NSCLC patients with pathogenic DNA-PK mutations. Therefore, future studies are necessary to investigate whether DNA-PK mutations would have similar clinical impacts on NSCLC patients when compared with those with *ATM*/*ATR* mutations. Further, the interaction between *ATM/ATR* and FA/HR mutations at cellular levels are unclear and may contribute to clinical outcomes; thus, mechanistic explorations are needed in future studies. Moreover, we acknowledge the potential contribution of tumor heterogeneity to our findings; however, the targeted NGS and RNA-seq used in this study are bulk sequencing methods, which have limited ability to differentiate cell subpopulations in tumor tissues. Future studies using other advanced sequencing technologies, such as single-cell DNA/RNA sequencing, hold great promise for revealing the contribution of tumor heterogeneity to the observations made in our study. Lastly, due to the limited time and resources of this project, there is no experimental or functional validation of the results using NSCLC cell lines; however, we believe this work has achieved its intended objective of uncovering the clinical relevance and molecular landscape of *ATM/ATR* mutations in NSCLC using largescale real-world patient genomics datasets, presenting a strong foundation for subsequent mechanistic investigations.

## Conclusions

In conclusion, we performed comprehensive genomic analysis to investigate the molecular and clinical significance of *ATM*/*ATR* pathogenic mutations in NSCLC, and we found *ATM*/*ATR*-mutated patients have a unique mutational profile and a specific enrichment of FA/HR aberrations, which might result in elevated nucleotide-level changes and poor prognosis. Our results proposed a novel DDR-driven tumorigenesis/progression model for NSCLC, and their reliance on aberrant DDR pathways implies the potential use of DDR inhibitors and/or immunotherapy to treat this subset of NSCLC patients.

### Supplementary Information

**Additional file 1: Figure S1.** The workflow of the study cohort and external cohort. **Figure S2.** PIKK pathogenic mutations were associated with higher mutational loads in NSCLC. (A) The TMB in PIKK-Mut and PIKK-WT NSCLC patients from the external cohort. (B) The comparison of TMB between different PIKK mutational status and histological subtypes in the external cohort. (C) The MSI in PIKK-Mut and PIKK-WT SCC patients from the study cohort. (D) The comparison of CIS stratified by both the PIKK mutational status and histological subtypes in the study cohort. (E) The oncoprint plot of NSCLC patients without any PIKK mutations from the study cohort. CNV, copy-number variation. **Figure S3.** The CNV enriched in PIKK-mutated patients. (A, B) The comparison of chromosome arm CNV (A) and gene-level CNV (B) between PIKK-mutated and PIKK-WT patients from the study cohort. The analyses were done using Fisher’s exact test, and all multiple comparisons were corrected by the Benjamini & Hochberg approach. **Figure S4.** The relationship between FA/HR mutations and demographic/clinical characteristics in the study cohort. (A-C) The age (A), sex (B), and histological subtype (C) differences in PIKK-mutated patients with different FA/HR mutational statuses. **Figure S5.** Patients with both PIKK and FA/HR mutations were associated with more nucleotide-level alterations. (A-C) The comparison of TMB (A), CIS (B), and MSI (C) in PIKK-mutated NSCLC patients with different FA/HR mutational statuses. **Figure S6.** PIKK and FA/HR co-mutations were associated with higher nucleotide-level changes and worse prognosis. (A, B) The comparison of TMB in NSCLC patients with different PIKK and FA (A) or HR (B) mutational statuses in the study cohort. (C, D) The comparison of CIS in NSCLC patients with different PIKK and FA (C) or HR (D) mutational statuses in the study cohort. (E, F) The comparison of MSI frequency in NSCLC patients with different PIKK and FA (E) or HR (F) mutational statuses in the study cohort. (G-I) The comparison of TMB in NSCLC patients with different PIKK and FA/HR mutational statuses in the external cohort. (J, K) Kaplan-Meier curve of overall survival in NSCLC patients from the external cohort in strata of different PIKK and FA (J) or HR (K) mutational statuses. **Figure S7.** CNV and pathways enriched in ATM-mutated versus ATR-mutated patients. (A, B) The gene-level CNV (A) and chromosome arm-level CNV (B) that were enriched in ATM-mutated or ATR-mutated patients. The analyses were done using Fisher’s exact test, and all multiple comparisons were corrected by the Benjamini & Hochberg approach. (C, D) The gene ontology analysis of mutated genes enriched in ATM-mutated patients (C) or ATR-mutated patients (D). **Figure S8.** The association of ATM/ATR and FA/HR co-mutations and various molecular and clinical characteristics. (A, B) The comparison of TMB in NSCLC patients from the study cohort who were stratified by different ATM/ATR and FA (A) or HR (B) mutational statuses. (C-E) The comparison of TMB in NSCLC patients from the external cohort who were stratified by different ATM/ATR and FA/HR mutational statuses. (F, G) The comparison of CIS in NSCLC patients from the study cohort who were stratified by different ATM/ATR and FA (F) or HR (G) mutational statuses. (H, I) The comparison of MSI frequency in NSCLC patients from the study cohort who were stratified by different ATM/ATR and FA (H) or HR (I) mutational statuses. (J) Kaplan-Meier curve of overall survival in NSCLC patients from the external cohort in strata of different ATM/ATR and FA mutational statuses. **Figure S9.** The proposed tumorigenesis model in DDR-mutation driven NSCLC tumors. **Table S1.** The ATM/ATR pathological mutations detected in the study cohort. **Table S2.** Demographic characteristics of the 499 NSCLC patients in the study cohort. **Table S3.** The ATM/ATR pathological mutations in the external cohort. **Table S4.** Demographic characteristics of the 2,727 NSCLC patients in the external cohort. **Table S5.** The list of mutated genes that were enriched in PIKK-mutated patients by at least 2 fold. **Table S6.** The list of mutated genes that were enriched in ATM or ATR by at least two folds.

## Data Availability

The human sequence data generated in this study are not publicly available due to patient privacy requirements but are available upon reasonable request from the corresponding author. Other datasets supporting the conclusions of this article are included within the article and its additional files.

## Abbreviations

ADC: Adenocarcinoma
ATM: Ataxia telangiectasia mutated protein
ATR: Ataxia telangiectasia and Rad3 related protein
CIS: Chromosome instability score
CNV: Copy-number variation
DDR: DNA damage response
FA: Fanconi anemia
FFPE: Formalin-fixed paraffin-embedded
GO: Gene ontology
HR: Homologous recombination
ICIs: Immune checkpoint inhibitors
KEGG: Kyoto encyclopedia of genes and genomes
LOH: Loss-of-heterozygosity
MSI: Microsatellite instabilities
Mut: Mutated
NGS: Next-generation sequencing
NSCLC: Non-small cell lung cancer
ORA: Over-representation analysis
OS: Overall survival
PARP: Poly(ADP-ribose) polymerase
PIKK: Phosphatidylinositol 3-kinase-related kinase
SCC: Squamous cell carcinoma
SNVs: Single-nucleotide variations
TCGA: The Cancer Genome Atlas
TMB: Tumor mutational burdens
Treg: Regulatory T cell
WT: Wild-type
