# Supplementary material for: The enrichment of Fanconi anemia/homologous recombination pathway aberrations in ATM/ATR-mutated NSCLC was accompanied by unique molecular features and poor prognosis
Source: J Transl Med. 2023 Dec 1;21:874. doi: 10.1186/s12967-023-04634-1 (PMC10690992; doi:10.1186/s12967-023-04634-1)
Supplement: Supplementary file 1 — Additional file 1: Figure S1. The workflow of the study cohort and external cohort. Figure S2. PIKK pathogenic mutations were associated with higher mutational loads in NSCLC. (A) The TMB in PIKK-Mut and PIKK-WT NSCLC patients from the external cohort. (B) The comparison of TMB between different PIKK mutational status and histological subtypes in the external cohort. (C) The MSI in PIKK-Mut and PIKK-WT SCC patients from the study cohort. (D) The comparison of CIS stratified by both the PIKK mutational status and histological subtypes in the study cohort. (E) The oncoprint plot of NSCLC patients without any PIKK mutations from the study cohort. CNV, copy-number variation. Figure S3. The CNV enriched in PIKK-mutated patients. (A, B) The comparison of chromosome arm CNV (A) and gene-level CNV (B) between PIKK-mutated and PIKK-WT patients from the study cohort. The analyses were done using Fisher’s exact test, and all multiple comparisons were corrected by the Benjamini & Hochberg approach. Figure S4. The relationship between FA/HR mutations and demographic/clinical characteristics in the study cohort. (A-C) The age (A), sex (B), and histological subtype (C) differences in PIKK-mutated patients with different FA/HR mutational statuses. Figure S5. Patients with both PIKK and FA/HR mutations were associated with more nucleotide-level alterations. (A-C) The comparison of TMB (A), CIS (B), and MSI (C) in PIKK-mutated NSCLC patients with different FA/HR mutational statuses. Figure S6. PIKK and FA/HR co-mutations were associated with higher nucleotide-level changes and worse prognosis. (A, B) The comparison of TMB in NSCLC patients with different PIKK and FA (A) or HR (B) mutational statuses in the study cohort. (C, D) The comparison of CIS in NSCLC patients with different PIKK and FA (C) or HR (D) mutational statuses in the study cohort. (E, F) The comparison of MSI frequency in NSCLC patients with different PIKK and FA (E) or HR (F) mutational statuses in the study cohort. [file 12967_2023_4634_MOESM1_ESM.docx]

**
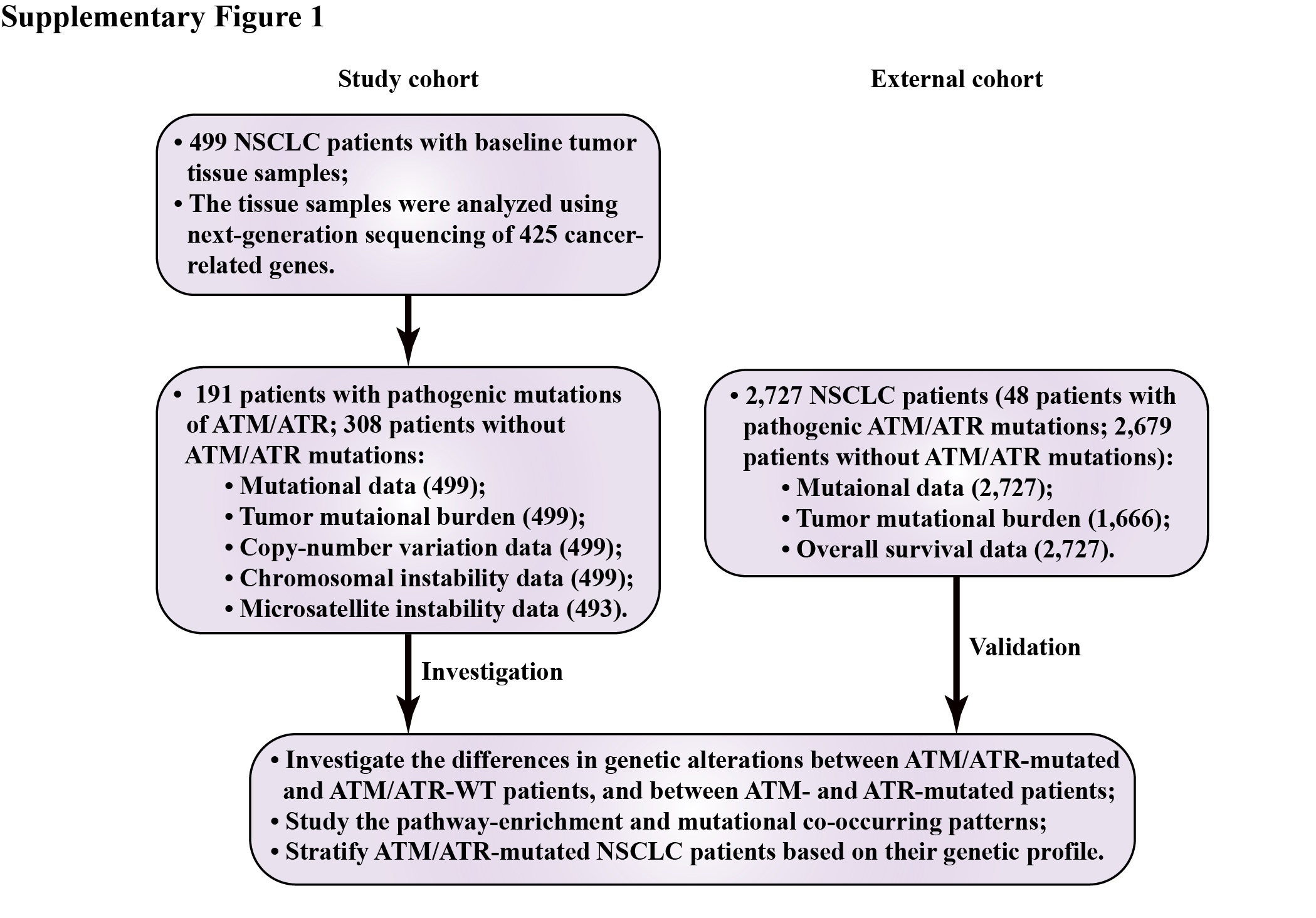
**

**Figure S1** The workflow of the study cohort and external cohort.

**
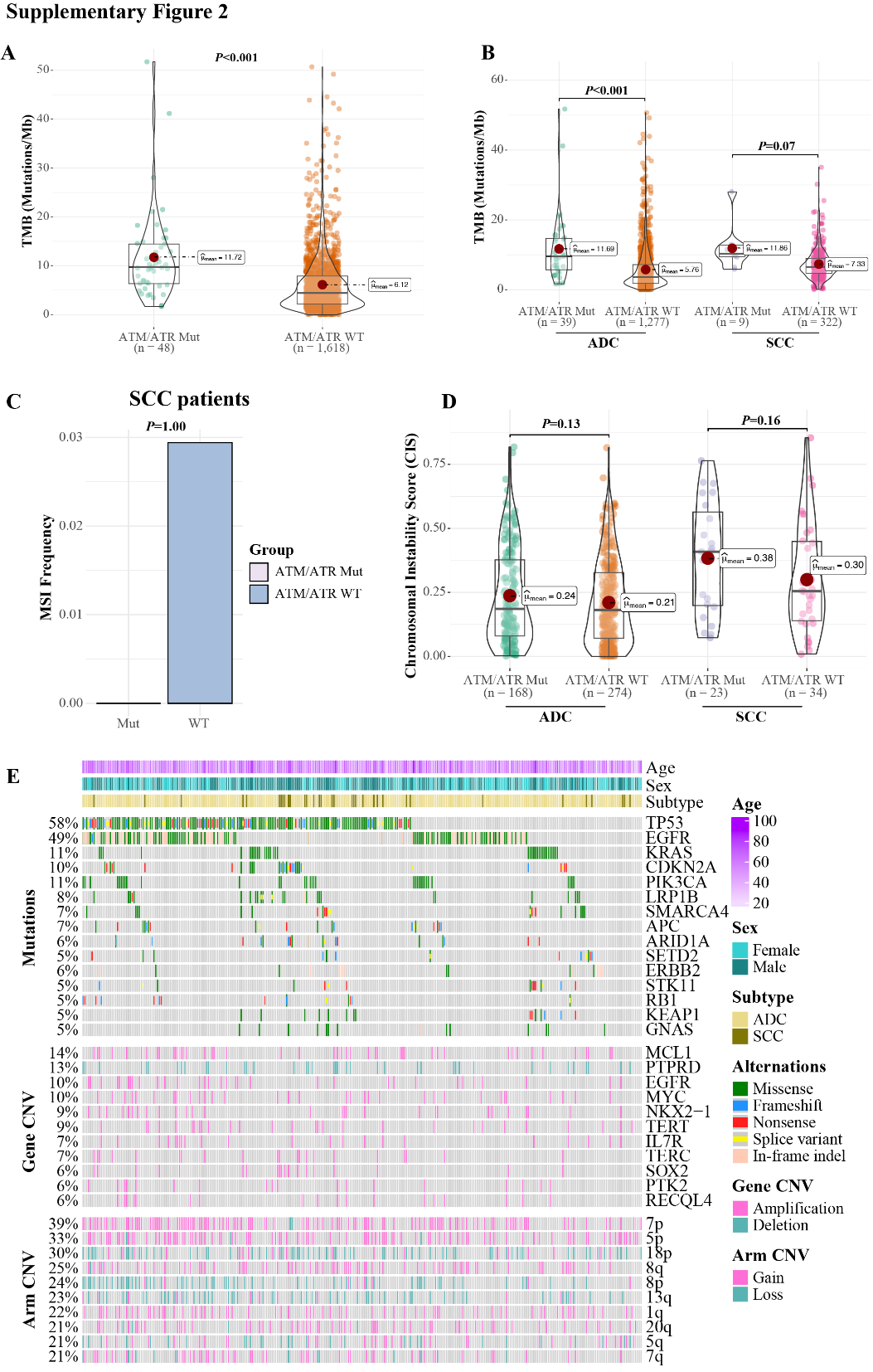
**

**Figure S2** PIKK pathogenic mutations were associated with higher mutational loads in NSCLC. (**A**) The TMB in PIKK-Mut and PIKK-WT NSCLC patients from the external cohort. (**B**) The comparison of TMB between different PIKK mutational status and histological subtypes in the external cohort. (**C**) The MSI in PIKK-Mut and PIKK-WT SCC patients from the study cohort. (**D**) The comparison of CIS stratified by both the PIKK mutational status and histological subtypes in the study cohort. (**E**) The oncoprint plot of NSCLC patients without any PIKK mutations from the study cohort. CNV, copy-number variation.

**
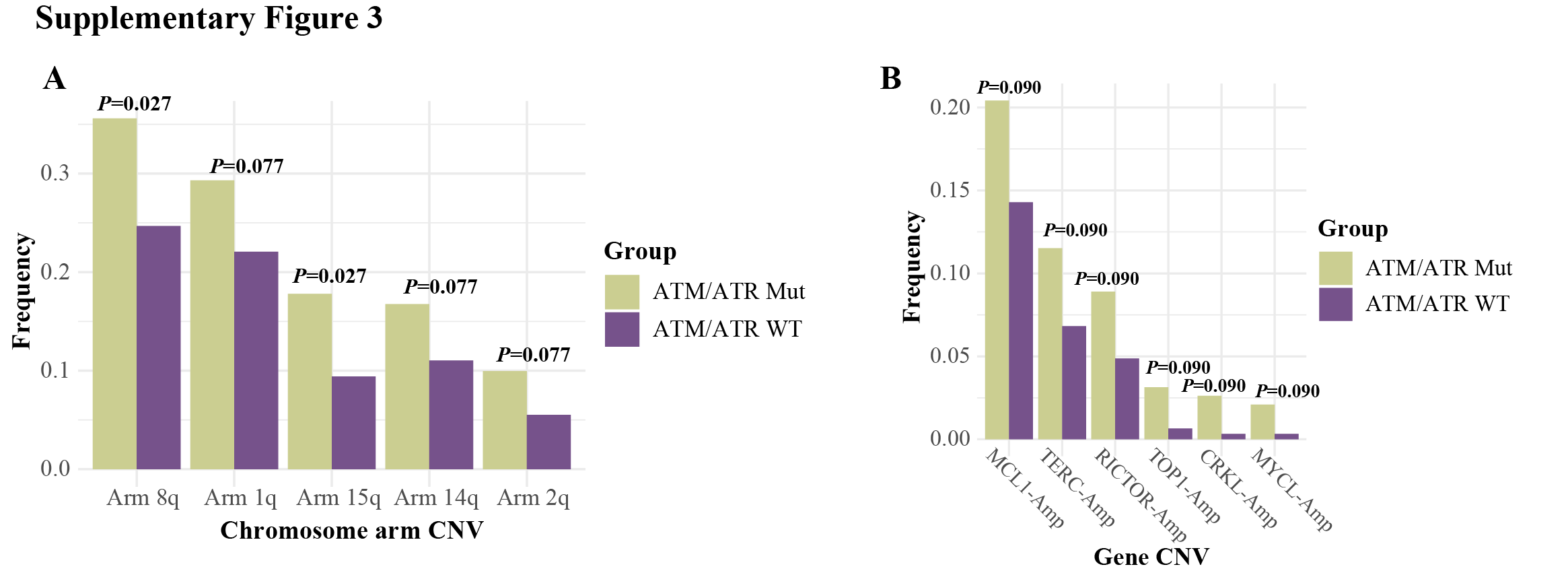
**

**Figure S3** The CNV enriched in PIKK-mutated patients. (**A**, **B**) The comparison of chromosome arm CNV (**A**) and gene-level CNV (**B**) between PIKK-mutated and PIKK-WT patients from the study cohort. The analyses were done using Fisher’s exact test, and all multiple comparisons were corrected by the Benjamini & Hochberg approach.

**
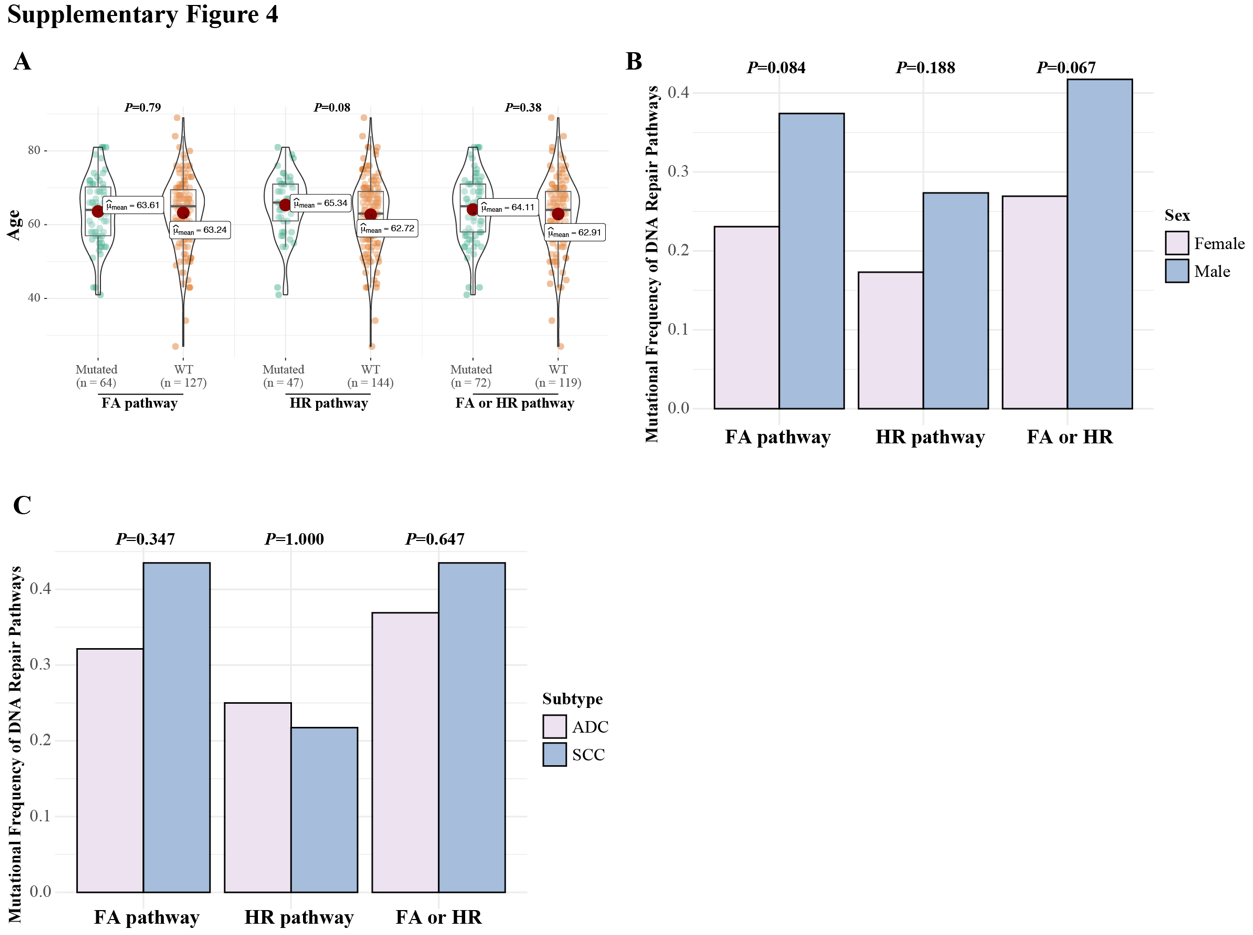
**

**Figure S4** The relationship between FA/HR mutations and demographic/clinical characteristics in the study cohort. (**A**-**C**) The age (**A**), sex (**B**), and histological subtype (**C**) differences in PIKK-mutated patients with different FA/HR mutational statuses.

**
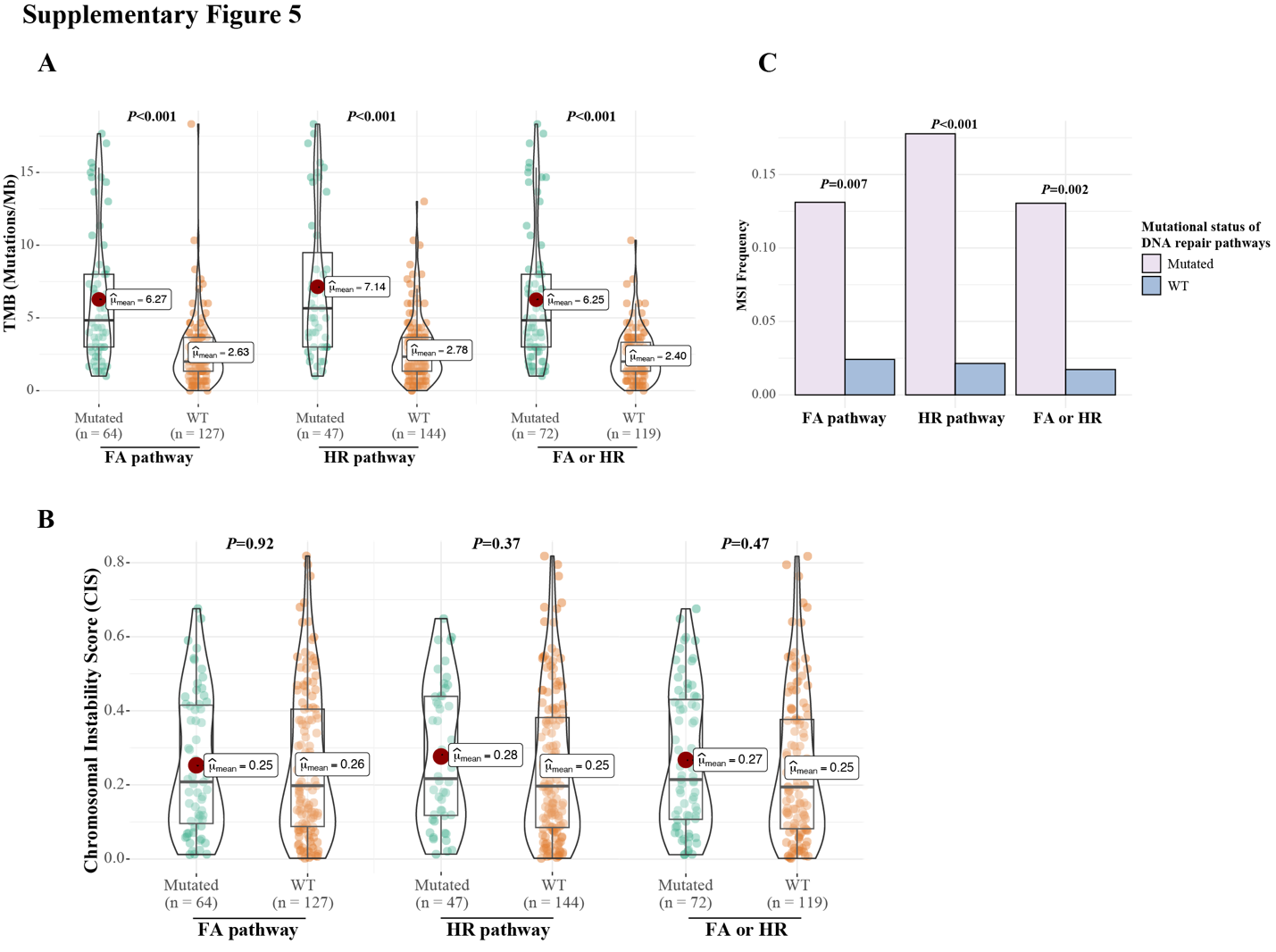
**

**Figure S5** Patients with both PIKK and FA/HR mutations were associated with more nucleotide-level alterations. (**A**-**C**) The comparison of TMB (**A**), CIS (**B**), and MSI (**C**) in PIKK-mutated NSCLC patients with different FA/HR mutational statuses.

**
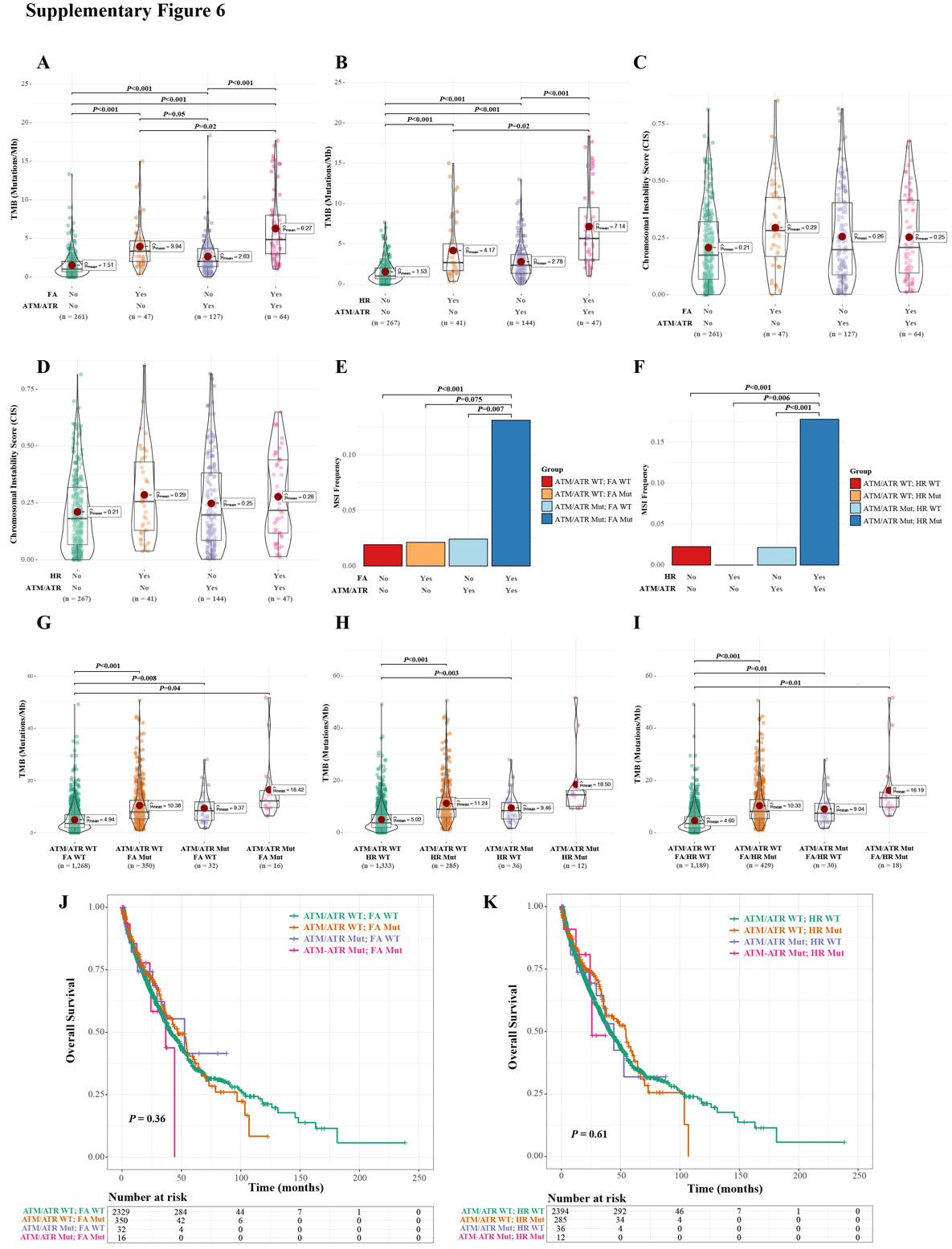
**

**Figure S6** PIKK and FA/HR co-mutations were associated with higher nucleotide-level changes and worse prognosis. (**A**, **B**) The comparison of TMB in NSCLC patients with different PIKK and FA (**A**) or HR (**B**) mutational statuses in the study cohort. (**C**, **D**) The comparison of CIS in NSCLC patients with different PIKK and FA (**C**) or HR (**D**) mutational statuses in the study cohort. (**E**, **F**) The comparison of MSI frequency in NSCLC patients with different PIKK and FA (**E**) or HR (**F**) mutational statuses in the study cohort. (**G**-**I**) The comparison of TMB in NSCLC patients with different PIKK and FA/HR mutational statuses in the external cohort. (**J**, **K**) Kaplan-Meier curve of overall survival in NSCLC patients from the external cohort in strata of different PIKK and FA (**J**) or HR (**K**) mutational statuses.

**
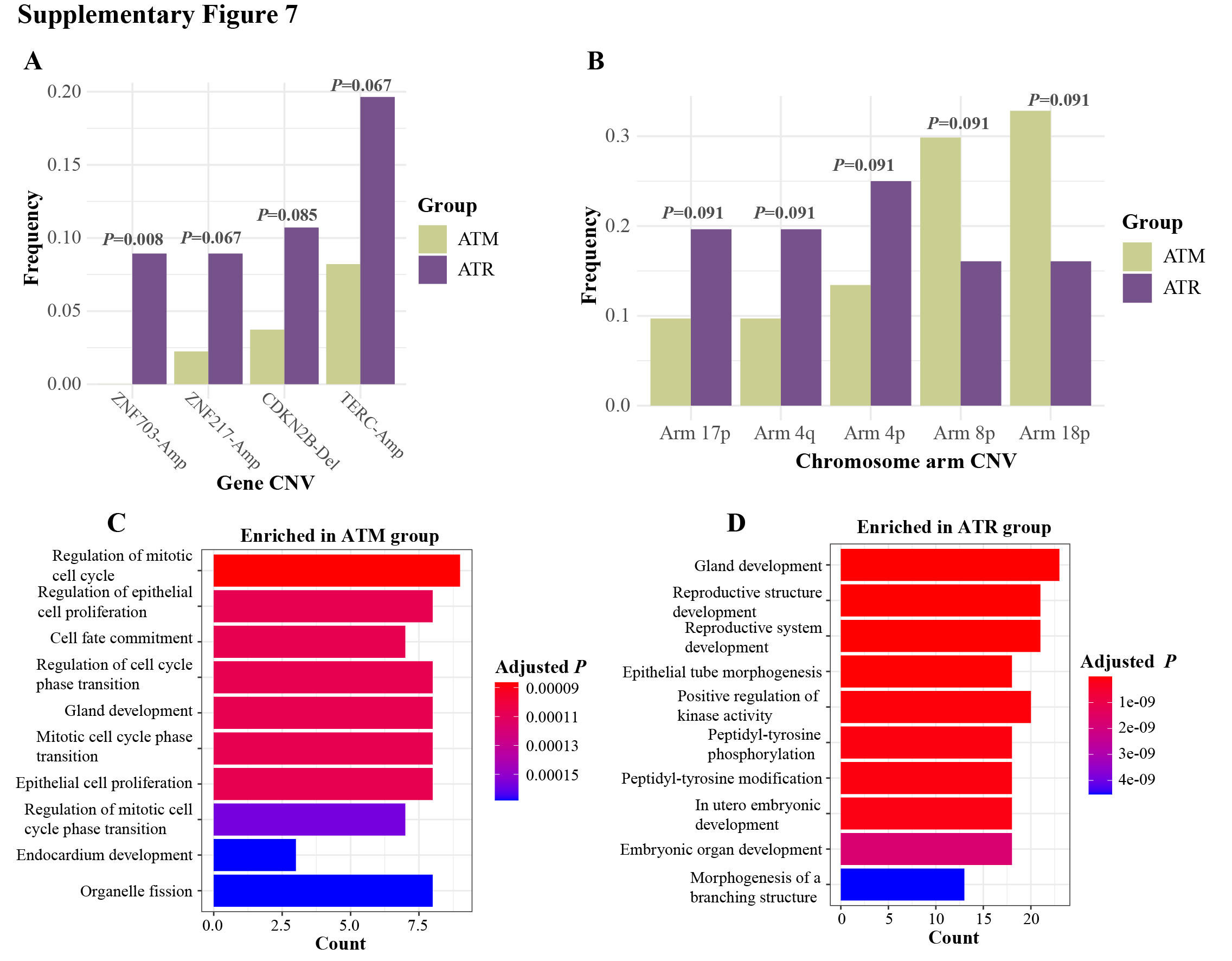
**

**Figure S7** CNV and pathways enriched in *ATM*-mutated versus *ATR*-mutated patients. (**A**, **B**) The gene-level CNV (**A**) and chromosome arm-level CNV (**B**) that were enriched in *ATM*-mutated or *ATR*-mutated patients. The analyses were done using Fisher’s exact test, and all multiple comparisons were corrected by the Benjamini & Hochberg approach. (**C**, **D**) The gene ontology analysis of mutated genes enriched in ATM-mutated patients (**C**) or ATR-mutated patients (**D**).

**
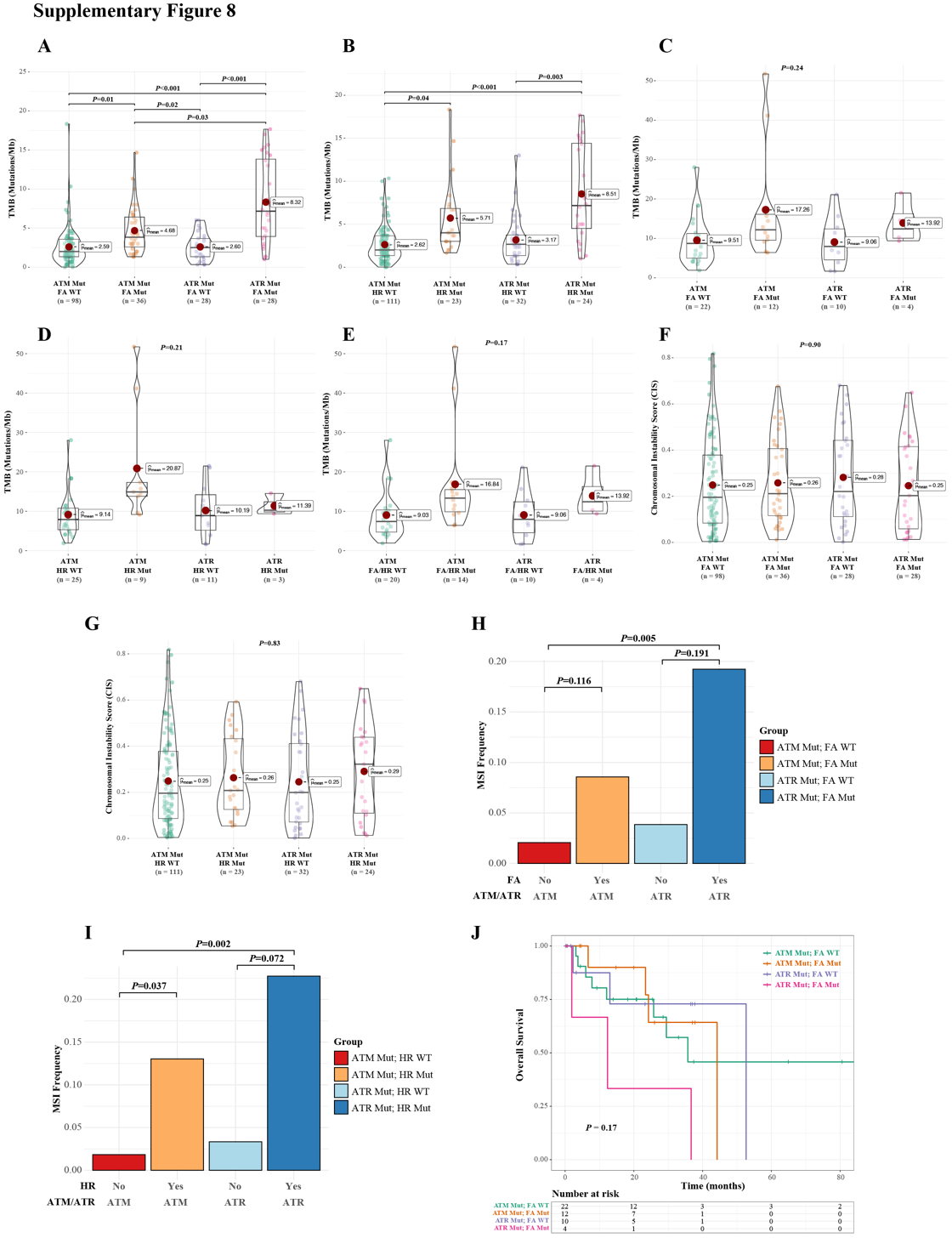
**

**Figure S8** The association of *ATM*/*ATR* and FA/HR co-mutations and various molecular and clinical characteristics. (**A**, **B**) The comparison of TMB in NSCLC patients from the study cohort who were stratified by different *ATM*/*ATR* and FA (**A**) or HR (**B**) mutational statuses. (**C**-**E**) The comparison of TMB in NSCLC patients from the external cohort who were stratified by different *ATM*/*ATR* and FA/HR mutational statuses. (**F**, **G**) The comparison of CIS in NSCLC patients from the study cohort who were stratified by different *ATM*/*ATR* and FA (**F**) or HR (**G**) mutational statuses. (**H**, **I**) The comparison of MSI frequency in NSCLC patients from the study cohort who were stratified by different *ATM*/*ATR* and FA (**H**) or HR (**I**) mutational statuses. (**J**) Kaplan-Meier curve of overall survival in NSCLC patients from the external cohort in strata of different *ATM*/*ATR* and FA mutational statuses.

**
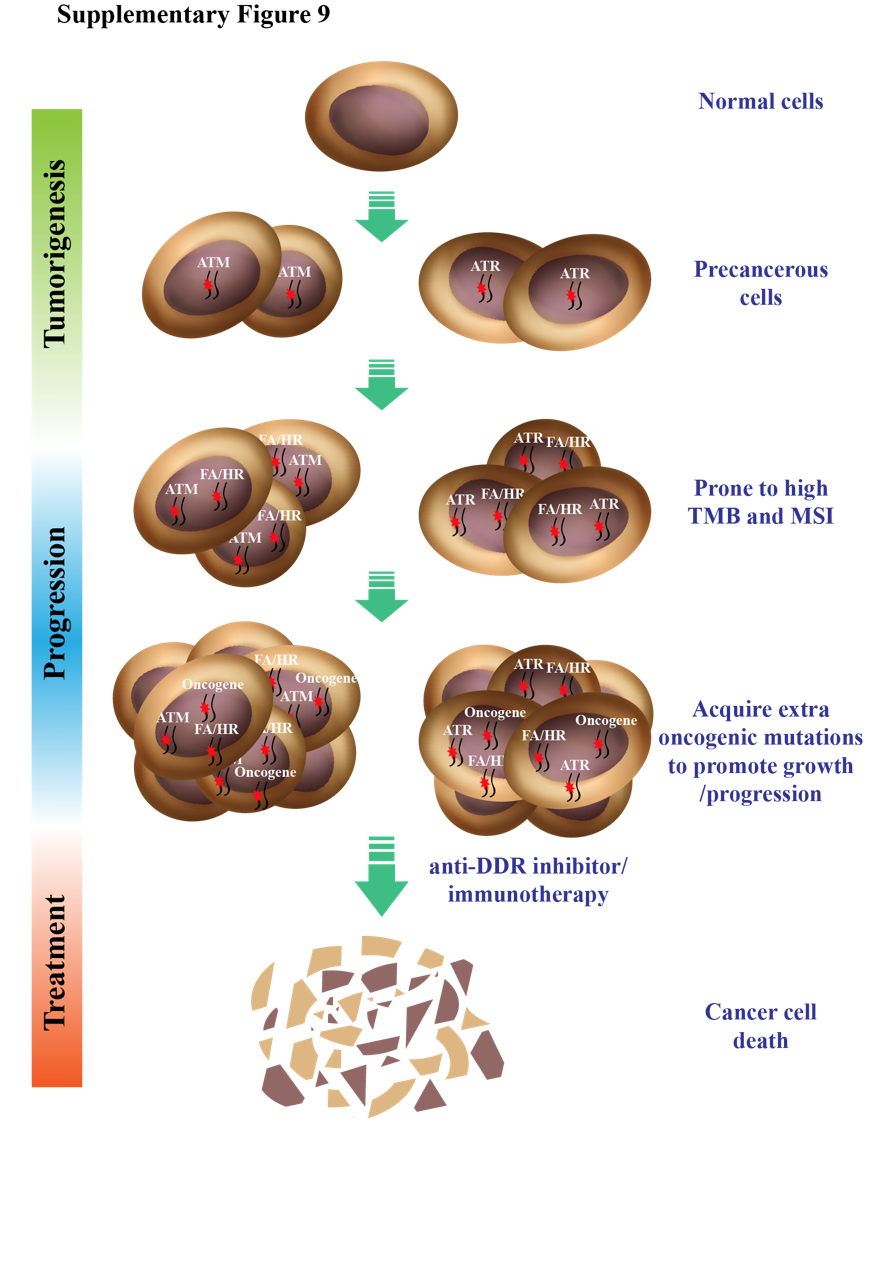
**

**Figure S9** The proposed tumorigenesis model in DDR-mutation driven NSCLC tumors.

**Table S1** The ATM/ATR pathological mutations detected in the study cohort.

| **Patient ID** | **Gene** | **Mutation** | **Variant**  **classification** | **ClinVar**  **classification** |
| --- | --- | --- | --- | --- |
| P1 | ATM | c.6399_6412delATCTCTAAGAGACA(p.S2134Rfs*7) | Frameshift |  |
| P2 | ATR | c.4387A>T(p.K1463*) | Nonsense |  |
| P3 | ATM | c.5692C>T(p.R1898*) | Nonsense |  |
| P4 | ATR | c.178_181delGACT(p.D60Lfs*6) | Frameshift |  |
| P5 | ATM | c.1039G>T(p.E347*) | Nonsense |  |
| P6 | ATR | c.2690_2691ins(149)(p.L897Ffs*92) | Frameshift |  |
| P7 | ATR | c.5173del(p.A1725Lfs*4) | Frameshift |  |
| P8 | ATR | c.1240C>T(p.Q414*) | Nonsense |  |
| P9 | ATM | c.8592C>G(p.Y2864*) | Nonsense |  |
| P10 | ATM | c.9139C>T(p.R3047*) | Nonsense | Pathogenic |
| P11 | ATR | c.6356T>A(p.L2119*) | Nonsense |  |
| P12 | ATM | c.4776+2T>C | Splice | Pathogenic |
| P13 | ATM | c.6214_6259delGGACTCTGCCATATTCTTTCCGTCTATTTAAAAGGATTGGATTATG(p.G2072Kfs*9) | Frameshift |  |
| P14 | ATM | c.8300delTinsCC(p.L2767Pfs*2) | Frameshift |  |
| P15 | ATM | c.1756G>T(p.E586*) | Nonsense |  |
| P16 | ATR | c.4097delC(p.P1366Qfs*4) | Frameshift |  |
| P17 | ATR | c.4888delA(p.T1630Pfs*4) | Frameshift |  |
| P18 | ATM | c.742C>T(p.R248*) | Nonsense | Pathogenic/Likely_pathogenic |
| P19 | ATM | c.521_530delTCTATCTGAA(p.L174Hfs*10) | Frameshift |  |
| P20 | ATR | c.2599delG(p.D867Ifs*3) | Frameshift |  |
| P21 | ATR | c.7789C>T(p.Q2597*) | Nonsense |  |
| P22 | ATR | c.3175G>T(p.E1059*) | Nonsense |  |
| P23 | ATM | c.1380_1381delGGinsTT(p.E461*) | Nonsense |  |
| P24 | ATM | c.3007C>T(p.Q1003*) | Nonsense |  |
| P25 | ATM | c.4772T>A(p.L1591*) | Nonsense |  |
| P26 | ATM | c.1464G>A(p.W488*) | Nonsense |  |
| P27 | ATR | c.1903C>T(p.R635*) | Nonsense |  |
| P28 | ATM | c.2089_2123delTTATCAGAACAGCTTCTGAATAATTACTCATCTGAinsATGAAAGTTCTTCTTTATCTTTTTGGT(p.L697Mfs*38) | Frameshift |  |
| P29 | ATR | c.3788dupA(p.I1264Dfs*14) | Frameshift |  |
| P30 | ATR | c.6409C>T(p.Q2137*) | Nonsense |  |
| P31 | ATR | c.2320dupA(p.I774Nfs*3) | Frameshift |  |
| P32 | ATM | c.8264_8268delATAAG(p.Y2755Cfs*12) | Frameshift | Pathogenic |
| P33 | ATR | c.684_687delACTT(p.L228Ffs*4) | Frameshift |  |
| P34 | ATR | c.3432delC(p.I1145Lfs*8) | Frameshift |  |
| P35 | ATM | c.1939G>T(p.E647*) | Nonsense | Pathogenic |
| P36 | ATR | c.4396C>T(p.Q1466*) | Nonsense |  |
| P37 | ATM | c.1236-2A>T | Splice | Pathogenic |
| P38 | ATR | c.4996A>T(p.K1666*) | Nonsense |  |
| P39 | ATM | c.67C>T(p.R23*) | Nonsense | Pathogenic |
| P40 | ATM | c.8645C>A(p.S2882*) | Nonsense |  |
| P41 | ATM | c.1027delG(p.E343Kfs*3) | Frameshift |  |
| P42 | ATM | c.4732C>T(p.Q1578*) | Nonsense | Likely_pathogenic |
| P43 | ATM | c.5216dupA(p.N1739Kfs*10) | Frameshift |  |
| P44 | ATR | c.2320dupA(p.I774Nfs*3) | Frameshift |  |
| P45 | ATR | c.3341dupT(p.S1115Ifs*3) | Frameshift |  |
| P46 | ATM | c.4145delC(p.P1382Hfs*4) | Frameshift |  |
| P47 | ATM | c.5121_5122insGGTAAATAATTATGTGAATTAATAAAAATATAAGAATTTTCATAAAGAA(p.L1708Gfs*3) | Nonsense |  |
| P48 | ATM | c.2854A>T(p.K952*) | Nonsense |  |
| P49 | ATM | c.1236-2A>T | Splice | Pathogenic |
| P50 | ATM | c.5200delG(p.V1734Lfs*4) | Frameshift |  |
| P51 | ATR | c.4826C>G(p.S1609*) | Nonsense |  |
| P52 | ATM | c.6615G>A(p.W2205*) | Nonsense |  |
| P53 | ATM | c.943_944delTT(p.L315Ifs*2) | Frameshift |  |
| P54 | ATM | c.1208C>G(p.S403*) | Nonsense |  |
| P55 | ATR | c.6815C>A(p.S2272*) | Nonsense |  |
| P56 | ATR | c.3386delG(p.G1129Afs*11) | Frameshift |  |
| P57 | ATM | c.6451A>T(p.R2151*) | Nonsense |  |
| P58 | ATM | c.642delC(p.K215Rfs*15) | Frameshift | Pathogenic |
| P59 | ATM | c.6757A>T(p.K2253*) | Nonsense |  |
| P60 | ATM | c.5492delG(p.C1831Lfs*3) | Frameshift |  |
| P61 | ATM | c.1234dupT(p.W412Lfs*18) | Frameshift |  |
| P62 | ATM | c.8720delC(p.P2907Lfs*31) | Frameshift |  |
| P62 | ATM | c.2461delA(p.S821Vfs*2) | Frameshift |  |
| P63 | ATR | c.5440dupA(p.R1814Kfs*8) | Frameshift |  |
| P64 | ATM | c.1236_1237insA(p.L413Tfs*17) | Frameshift |  |
| P65 | ATM | c.8122G>A(p.D2708N) | Missense | Likely_pathogenic |
| P66 | ATM | c.802C>T(p.Q268*) | Nonsense | Pathogenic/Likely_pathogenic |
| P67 | ATM | c.757G>T(p.E253*) | Nonsense |  |
| P68 | ATM | c.9019delG(p.E3007Nfs*4) | Frameshift |  |
| P69 | ATR | c.7792C>T(p.R2598*) | Nonsense |  |
| P70 | ATM | c.7777C>T(p.Q2593*) | Nonsense |  |
| P71 | ATM | c.4765delT(p.S1589Hfs*12) | Frameshift |  |
| P72 | ATR | c.3043delC(p.R1015Efs*3) | Frameshift |  |
| P73 | ATM | c.4282G>T(p.E1428*) | Nonsense |  |
| P74 | ATM | c.3077+1delG | Frameshift |  |
| P75 | ATM | c.7886dupT(p.L2630Ifs*26) | Frameshift |  |
| P76 | ATM | c.1898+1delG | Frameshift |  |
| P77 | ATM | c.4169_4178delTTGCCTATAT(p.F1390Sfs*9) | Frameshift |  |
| P78 | ATM | c.6268_6280delGACTGGTGTCCTG(p.D2090Nfs*2) | Frameshift |  |
| P79 | ATM | c.465C>G(p.Y155*) | Nonsense |  |
| P80 | ATR | c.1038delG(p.L346Ffs*27) | Frameshift |  |
| P81 | ATM | c.7542T>A(p.Y2514*) | Nonsense |  |
| P81 | ATM | c.5823dupT(p.A1942Cfs*23) | Frameshift |  |
| P82 | ATM | c.6661G>T(p.E2221*) | Nonsense |  |
| P83 | ATM | c.8287C>T(p.R2763*) | Nonsense | Pathogenic |
| P84 | ATR | c.5440dupA(p.R1814Kfs*8) | Frameshift |  |
| P85 | ATM | c.7886_7890delTATTA(p.I2629Sfs*25) | Frameshift | Pathogenic |
| P86 | ATM | c.2866G>T(p.G956*) | Nonsense |  |
| P87 | ATM | c.1236-2A>T | Splice | Pathogenic |
| P88 | ATM | c.6572+46_6572+47ins(147) | Nonsense |  |
| P89 | ATR | c.244dupA(p.M82Nfs*10) | Frameshift |  |
| P90 | ATM | c.1311delG(p.M437Ifs*36) | Frameshift |  |
| P91 | ATM | c.1102C>T(p.Q368*) | Nonsense |  |
| P92 | ATR | c.4417G>T(p.G1473*) | Nonsense |  |
| P93 | ATM | c.8494C>T(p.R2832C) | Missense | Pathogenic/Likely_pathogenic |
| P94 | ATM | c.8230G>T(p.E2744*) | Nonsense |  |
| P95 | ATM | c.5697C>A(p.C1899*) | Nonsense | Pathogenic |
| P96 | ATR | c.7138delG(p.V2380*) | Frameshift |  |
| P97 | ATM | c.7533_7542delTCCAACATAT(p.P2512Nfs*17) | Frameshift |  |
| P98 | ATM | c.8293G>A(p.G2765S) | Missense | Pathogenic/Likely_pathogenic |
| P99 | ATM | c.1071delT(p.F357Lfs*33) | Frameshift |  |
| P100 | ATM | c.1236-2A>T | Splice | Pathogenic |
| P101 | ATR | c.3220G>T(p.G1074*) | Nonsense |  |
| P102 | ATR | c.2188G>T(p.E730*) | Nonsense |  |
| P103 | ATR | c.5383G>T(p.G1795*) | Nonsense |  |
| P104 | ATR | c.5293G>T(p.E1765*) | Nonsense |  |
| P105 | ATR | c.2320dupA(p.I774Nfs*3) | Frameshift |  |
| P106 | ATM | c.601C>T(p.Q201*) | Nonsense | Likely_pathogenic |
| P107 | ATM | c.8545C>T(p.R2849*) | Nonsense | Pathogenic |
| P108 | ATM | c.7149_7150delinsG(p.M2384*) | Frameshift |  |
| P109 | ATM | c.447delT(p.L150Ffs*3) | Frameshift |  |
| P109 | ATM | c.5044G>C(p.D1682H) | Missense | Pathogenic |
| P110 | ATM | c.709dupA(p.T237Nfs*17) | Frameshift |  |
| P111 | ATM | c.583_584delACinsT(p.T195Sfs*11) | Frameshift |  |
| P112 | ATM | c.5395_5405delAGTGAAAATCA(p.S1799*) | Frameshift |  |
| P113 | ATM | c.2209G>T(p.E737*) | Nonsense |  |
| P114 | ATR | c.4154delC(p.T1385Mfs*3) | Frameshift |  |
| P115 | ATR | c.4082T>A(p.L1361*) | Nonsense |  |
| P116 | ATR | c.2320dupA(p.I774Nfs*3) | Frameshift |  |
| P117 | ATM | c.6049_6052delAGTT(p.S2017Cfs*29) | Frameshift |  |
| P118 | ATM | c.8325delC(p.I2776Lfs*30) | Frameshift | Pathogenic/Likely_pathogenic |
| P119 | ATR | c.2320dupA(p.I774Nfs*3) | Frameshift |  |
| P120 | ATM | c.8293G>A(p.G2765S) | Missense | Pathogenic/Likely_pathogenic |
| P121 | ATM | c.700delG(p.A234Qfs*21) | Frameshift |  |
| P122 | ATM | c.2548G>T(p.E850*) | Nonsense | Pathogenic |
| P123 | ATM | c.2212G>T(p.E738*) | Nonsense |  |
| P124 | ATM | c.1597A>T(p.R533*) | Nonsense |  |
| P125 | ATM | c.1093G>T(p.E365*) | Nonsense | Pathogenic |
| P126 | ATM | c.8398C>T(p.Q2800*) | Nonsense |  |
| P127 | ATM | c.3154-1G>T | Splice | Likely_pathogenic |
| P128 | ATM | c.7189C>T(p.Q2397*) | Nonsense | Pathogenic |
| P129 | ATM | c.3231delT(p.A1079Lfs*30) | Frameshift |  |
| P130 | ATM | c.3176_3194delCCATTCTTAATGTAATGGG(p.A1059Efs*6) | Frameshift |  |
| P131 | ATR | c.4264C>T(p.Q1422*) | Nonsense |  |
| P132 | ATR | c.3883G>T(p.E1295*) | Nonsense |  |
| P133 | ATM | c.5697C>A(p.C1899*) | Nonsense | Pathogenic |
| P134 | ATM | c.1249_1265delACCCAATTAATATCAAA(p.T417Vfs*7) | Frameshift |  |
| P135 | ATM | c.1750C>T(p.Q584*) | Nonsense |  |
| P136 | ATM | c.2548G>T(p.E850*) | Nonsense | Pathogenic |
| P137 | ATM | c.7267G>T(p.E2423*) | Nonsense |  |
| P138 | ATM | c.6725C>G(p.S2242*) | Nonsense |  |
| P139 | ATM | c.5908C>T(p.Q1970*) | Nonsense | Pathogenic |
| P140 | ATR | c.361A>T(p.K121*) | Nonsense |  |
| P141 | ATM | c.3145_3146insC(p.L1049Sfs*3) | Frameshift |  |
| P142 | ATM | c.6825_6826del(p.F2276Sfs*3) | Frameshift |  |
| P143 | ATM | c.7708G>T(p.E2570*) | Nonsense | Pathogenic/Likely_pathogenic |
| P144 | ATM | c.635_636insG(p.F212Lfs*42) | Frameshift |  |
| P145 | ATR | c.2770del(p.S924Vfs*15) | Frameshift |  |
| P145 | ATM | c.7476_7477insA(p.E2493Rfs*7) | Frameshift |  |
| P145 | ATM | c.7477delinsAA(p.E2493Kfs*7) | Frameshift |  |
| P146 | ATM | c.3089dup(p.E1031Gfs*17) | Frameshift |  |
| P147 | ATR | c.512G>A(p.W171*) | Nonsense |  |
| P148 | ATM | c.3402+2T>C | Splice | Likely_pathogenic |
| P149 | ATM | c.3424G>T(p.E1142*) | Nonsense |  |
| P150 | ATM | c.6272G>A(p.W2091*) | Nonsense | Pathogenic |
| P151 | ATM | c.1077del(p.D360Ifs*30) | Frameshift |  |
| P152 | ATM | c.1102C>T(p.Q368*) | Nonsense |  |
| P153 | ATM | c.1236-1G>A | Splice | Likely_pathogenic |
| P154 | ATM | c.2638+1G>T | Splice | Likely_pathogenic |
| P155 | ATR | c.1602G>A(p.W534*) | Nonsense |  |
| P156 | ATM | c.8157_8166del(p.D2721Kfs*27) | Frameshift |  |
| P157 | ATR | c.6313delinsAA(p.E2105Kfs*14) | Frameshift |  |
| P158 | ATM | c.8283_8284del(p.Q2762Afs*6) | Frameshift | Pathogenic |
| P159 | ATM | c.4292_4296del(p.N1431Ifs*2) | Frameshift |  |
| P160 | ATR | c.6373_6382del(p.V2125Sfs*6) | Frameshift |  |
| P161 | ATM | c.7261A>T(p.K2421*) | Nonsense |  |
| P162 | ATM | c.7413_7428del(p.I2471Mfs*28) | Frameshift |  |
| P163 | ATM | c.8929G>T(p.E2977*) | Nonsense |  |
| P164 | ATR | c.6265C>T(p.R2089*) | Nonsense |  |
| P165 | ATM | c.8293G>A(p.G2765S) | Missense | Pathogenic/Likely_pathogenic |
| P166 | ATM | c.2340del(p.M780Ifs*8) | Frameshift |  |
| P167 | ATM | c.5971G>T(p.E1991*) | Nonsense | Pathogenic |
| P168 | ATM | c.6715del(p.M2239Wfs*18) | Frameshift |  |
| P169 | ATM | c.5623C>T(p.R1875*) | Nonsense | Pathogenic |
| P170 | ATR | c.5020G>T(p.G1674*) | Nonsense |  |
| P171 | ATM | c.901+1G>T | Splice | Pathogenic/Likely_pathogenic |
| P172 | ATM | c.9139C>T(p.R3047*) | Nonsense | Pathogenic |
| P173 | ATM | c.1426C>T(p.Q476*) | Nonsense |  |
| P174 | ATM | c.2193C>A(p.Y731*) | Nonsense | Pathogenic |
| P175 | ATM | c.967A>G(p.I323V) | Missense | Pathogenic/Likely_pathogenic |
| P176 | ATM | c.7191del(p.Q2397Hfs*9) | Frameshift |  |
| P177 | ATR | c.5485G>T(p.E1829*) | Nonsense |  |
| P178 | ATM | c.7181C>T(p.S2394L) | Missense | Likely_pathogenic |
| P179 | ATR | c.1227_1236del(p.E409Dfs*27) | Frameshift |  |
| P180 | ATR | c.6265C>T(p.R2089*) | Nonsense |  |
| P181 | ATM | c.1750C>T(p.Q584*) | Nonsense | Pathogenic |
| P182 | ATM | c.4177_4181del(p.I1393Qfs*4) | Frameshift |  |
| P183 | ATM | c.3993+1G>T | Splice | Likely_pathogenic |
| P184 | ATM | c.2921+1G>T | Splice | Pathogenic/Likely_pathogenic |
| P185 | ATR | c.1303_1312del(p.S435*) | Frameshift |  |
| P186 | ATM | c.6998_6999del(p.T2333Ifs*39) | Frameshift |  |
| P187 | ATR | c.5656C>T(p.R1886*) | Nonsense |  |
| P188 | ATM | c.1021_1022del(p.V341Qfs*6) | Frameshift |  |
| P189 | ATM | c.4741del(p.I1581Sfs*20) | Frameshift |  |
| P189 | ATM | c.4741_4742delinsG(p.I1581Afs*20) | Frameshift |  |
| P190 | ATM | c.4043_4046del(p.L1348*) | Frameshift |  |
| P191 | ATR | c.5413G>T(p.G1805*) | Nonsense |  |

**Table S2** Demographic characteristics of the 499 NSCLC patients in the study cohort.

| **Characteristics** | **With ATM/ATR pathological mutations (n=191)** | **Without ATM/ATR mutations (n=308)** | ***P* values** |
| --- | --- | --- | --- |
| Age (median, range) | 64 (27-89) | 60 (28-89) | <0.001 |
| Sex |  |  | <0.001 |
| Male | 139 (72.8%) | 172 (55.8%) |  |
| Female | 52 (27.2%) | 136 (44.2%) |  |
| Histologic subtype |  |  | 0.773 |
| ADC | 168 (88.0%) | 274 (89.0%) |  |
| SCC | 23 (12.0%) | 34 (11.0%) |  |

**Table S3** The ATM/ATR pathological mutations in the external cohort.

| **Patient ID** | **Gene** | **Mutation** | **Variant classification** |
| --- | --- | --- | --- |
| E1 | ATR | p.E7Dfs*21 | Frameshift |
| E2 | ATR | p.E1329* | Nonsense |
| E3 | ATM | p.E1822* | Nonsense |
| E4 | ATR | p.W102* | Nonsense |
| E5 | ATM | p.G2369* | Nonsense |
| E6 | ATM | p.E1313Gfs*36 | Frameshift |
| E7 | ATM | p.G956* | Nonsense |
| E7 | ATM | p.G956* | Nonsense |
| E8 | ATM | p.Q1084* | Nonsense |
| E9 | ATM | p.G2536Efs*6 | Frameshift |
| E10 | ATR | p.R1814Kfs*8 | Frameshift |
| E11 | ATM | p.Q445Lfs*42 | Frameshift |
| E12 | ATM | p.Q2615* | Nonsense |
| E13 | ATR | p.S1372* | Nonsense |
| E14 | ATM | p.G873* | Nonsense |
| E15 | ATR | p.L537* | Nonsense |
| E16 | ATM | p.D2569* | Frameshift |
| E17 | ATM | p.I1581Nfs*5 | Frameshift |
| E18 | ATM | p.Q1636Rfs*10 | Frameshift |
| E19 | ATM | p.Y889Ifs*10 | Frameshift |
| E20 | ATM | p.E871* | Nonsense |
| E21 | ATM | p.E1940* | Nonsense |
| E22 | ATM | p.Q1225* | Nonsense |
| E23 | ATM | p.E1979* | Nonsense |
| E24 | ATM | p.S2882* | Nonsense |
| E25 | ATR | p.S2207Ifs*15 | Frameshift |
| E26 | ATM | p.E3015* | Nonsense |
| E27 | ATR | p.K343* | Nonsense |
| E28 | ATM | p.S751Ffs*14 | Frameshift |
| E28 | ATM | p.S1487* | Nonsense |
| E29 | ATM | p.Q628* | Nonsense |
| E30 | ATM | p.E770* | Nonsense |
| E31 | ATR | p.E833* | Nonsense |
| E32 | ATR | p.S1616* | Nonsense |
| E33 | ATR | p.G736* | Nonsense |
| E34 | ATM | p.I1525Nfs*6 | Frameshift |
| E35 | ATM | p.K1196* | Nonsense |
| E36 | ATM | p.R248* | Nonsense |
| E37 | ATM | p.C819Lfs*4 | Frameshift |
| E38 | ATM | p.R1437* | Nonsense |
| E39 | ATR | p.S508Lfs*5 | Frameshift |
| E40 | ATM | p.R250Sfs*3 | Frameshift |
| E40 | ATM | p.L2006* | Frameshift |
| E41 | ATM | p.T1029Qfs*10 | Frameshift |
| E42 | ATM | p.Q65* | Nonsense |
| E43 | ATR | p.I97* | Frameshift |
| E44 | ATM | p.L427* | Nonsense |
| E45 | ATM | p.L210Wfs*20 | Frameshift |
| E46 | ATM | p.Q1116Sfs*10 | Frameshift |
| E47 | ATR | p.Q2085* | Nonsense |
| E48 | ATM | p.Q2277* | Nonsense |

**Table S4** Demographic characteristics of the 2,727 NSCLC patients in the external cohort.

| **Characteristics** | **With ATM/ATR pathological mutations (n=48)** | **Without ATM/ATR mutations (n=2,679)** | ***P* values** |
| --- | --- | --- | --- |
| Age (mean, range) | 65 (44-84) | 67 (25-93) | 0.303 |
| Sex |  |  | 0.306 |
| Male | 18 (37.5%) | 1,223 (45.6%) |  |
| Female | 30 (62.5%) | 1,446 (54.0%) |  |
| NA | 0 (0.0%) | 10 (0.4%) |  |
| Histologic subtype |  |  | 0.543 |
| ADC | 39 (81.3%) | 2,176 (81.2%) |  |
| SCC | 9 (18.7%) | 394 (14.7%) |  |
| NA | 0 (0.0%) | 109 (4.1%) |  |

**Table S5** The list of mutated genes that were enriched in PIKK-mutated patients by at least 2 fold.

| ABCB1 | LZTR1 | EPHA2 | PKHD1 | CDC73 | NRAS | GNAQ | SMO |
| --- | --- | --- | --- | --- | --- | --- | --- |
| ADGRB3 | MAP2K4 | EPHA5 | POLD1 | CDH1 | NSD1 | GRIN2A | SOX2 |
| ALK | MAP3K4 | ERCC2 | POT1 | CDK12 | NTRK1 | GRM8 | SPRY4 |
| ASCL4 | MCL1 | ERCC5 | PRF1 | CDK8 | NTRK3 | GSTM5 | SRC |
| AURKB | MDM2 | EXT1 | PRKCI | CDKN1B | PALB2 | HDAC2 | STAG2 |
| AXIN2 | MECOM | FANCA | PRSS1 | CEBPA | PALLD | HNF1A | STK11 |
| B2M | MEN1 | FANCC | PTCH1 | CEP57 | PARK2 | IDH1 | TERT |
| BAP1 | MITF | FANCE | PTPN11 | CHD4 | PARP1 | IDH2 | TGFBR2 |
| BARD1 | MLH1 | FANCF | RAD54L | CHD8 | PAX5 | IGF1R | TNFRSF11A |
| BLM | MLH3 | FANCG | RET | CHEK1 | PBRM1 | IGF2 | TOP2A |
| BMPR1A | MPL | FANCL | ROS1 | CHEK2 | PDCD1 | INPP4B | TSC1 |
| BRCA1 | MSH2 | FBXW7 | RPTOR | CRKL | PDE11A | KDM5A | TTF1 |
| BRCA2 | MSH6 | FGFR2 | RRM1 | CSF1R | PDGFRA | KDR | WAS |
| BRD4 | MYC | FGFR4 | RUNX1 | CYLD | PDGFRB | KLLN | WISP3 |
| BRIP1 | NBN | FH | RUNX1T1 | CYP2C19 | PDK1 | KMT2C | WRN |
| BTK | NF1 | FLT1 | SETBP1 | DDR2 | PIK3R2 | KRAS | WT1 |
| C11orf30 | NOTCH1 | GATA3 | SGK1 | DENND1A | PKD1 | LHCGR | XPC |
| CCNE1 | NPM1 | GATA4 | SMAD3 | EPAS1 | PKD2 | ZNF703 |  |

**Table S6** The list of mutated genes that were enriched in ATM or ATR by at least two folds.

| **Category** | **Gene list** |
| --- | --- |
| Enriched in ATM than ATR group | AURKB B2M BUB1B CDC73 CDKN1C CHEK2 CYP2C19 DENND1A DLL3 EPHA5 ERBB4 FANCD2 HDAC9 JAK3 KDR KMT2C KRAS MCL1 MECOM MET MLH3 MYC NRG1 PTPN13 RAD54L RPTOR RRM1 SMAD4 STAT3 STK11 UGT1A1 |
| Enriched in ATR than ATM group | AMER1 AXIN2 BRAF BRCA1 BRCA2 BRIP1 BTK C11orf30 CBL CBLB CDH1 CDK8 CEP57 CHD4 CHD8 CREBBP CSF1R CYLD DAXX DDR2 DICER1 DNMT3A ERBB2 ERCC2 FANCG FBXW7 FGFR1 FGFR2 FLT3 GATA6 GNAQ GNAS HGF IGF2 JAK1 KDM5A KMT2B MED12 MLH1 MSH2 MTOR NFE2L2 NKX2-1 NOTCH1 NOTCH2 NPM1 NRAS NSD1 NTRK1 PALB2 PALLD PAX5 PDCD1 PDK1 PGR PKD1 PKD2 PLCB4 PMS1 POLD1 POLE POLH PRKCI PRSS1 PTCH1 RET SETD2 SMAD3 SMARCA4 SPRY4 SRC TEK TET2 TGFBR2 WISP3 WT1 AKT1 BLM BMPR1A CTCF DPYD ERCC3 FANCE FOXA1 IDH2 KLLN PARP1 RAD51D RARA RUNX1 STAG2 TAP2 |
